# Terazosin, a repurposed GPR119 agonist, ameliorates mitophagy and β‐cell function in NAFPD by inhibiting MST1‐Foxo3a signalling pathway

**DOI:** 10.1111/cpr.13764

**Published:** 2024-10-16

**Authors:** Chenglei Zhang, Jiarui Li, Lijuan Wang, Jie Ma, Xin Li, Yuanyuan Wu, Yanru Ren, Yanhui Yang, Hui Song, Jianning Li, Yi Yang

**Affiliations:** ^1^ School of Basic Medical Sciences Ningxia Medical University Yinchuan Ningxia China; ^2^ Medical Laboratory General Hospital of Ningxia Medical University Yinchuan Ningxia China; ^3^ Department of Endocrinology General Hospital of Ningxia Medical University Yinchuan Ningxia China; ^4^ Department of Oncology, Cancer Hospital General Hospital of Ningxia Medical University Yinchuan Ningxia China; ^5^ Day‐Care Unit General Hospital of Ningxia Medical University Yinchuan Ningxia China

## Abstract

GPR119 agonists are being developed to safeguard the function of pancreatic β‐cells, especially in the context of non‐alcoholic fatty pancreas disease (NAFPD) that is closely associated with β‐cell dysfunction. This study aims to employ a drug repurposing strategy to screen GPR119 agonists and explore their potential molecular mechanisms for enhancing β‐cell function in the context of NAFPD. MIN6 cells were stimulated with palmitic acid (PA), and a NAFPD model was established in GPR119^−/−^ mice fed with a high‐fat diet (HFD). Terazosin, identified through screening, was utilized to assess its impact on enhancing β‐cell function via the MST1‐Foxo3a pathway and mitophagy. Terazosin selectively activated GPR119, leading to increased cAMP and ATP synthesis, consequently enhancing insulin secretion. Terazosin administration improved high blood glucose, obesity, and impaired pancreatic β‐cell function in NAFPD mice. It inhibited the upregulation of MST1‐Foxo3a expression in pancreatic tissue and enhanced damaged mitophagy clearance, restoring autophagic flux, and improving mitochondrial quantity and structure in β‐cells. Nevertheless, GPR119 deficiency negated the positive impact of terazosin on pancreatic β‐cell function in NAFPD mice and abolished its inhibitory effect on the MST1‐Foxo3a pathway. Terazosin activates GPR119 on the surface of pancreatic β‐cells, enhancing mitophagy and alleviating β‐cell dysfunction in the context of NAFPD by suppressing the MST1‐Foxo3a signalling pathway. Terazosin could be considered a priority treatment for patients with concomitant NAFPD and hypertension.

## Graphical abstract

Antihypertensive drug terazosin, a repurposed GPR119 agonist, demonstrates potential in addressing β‐cell dysfunction associated with NAFPD. Terazosin enhances mitophagy and alleviates β‐cell dysfunction in NAFPD by inhibiting the MST1‐Foxo3a signalling pathway.

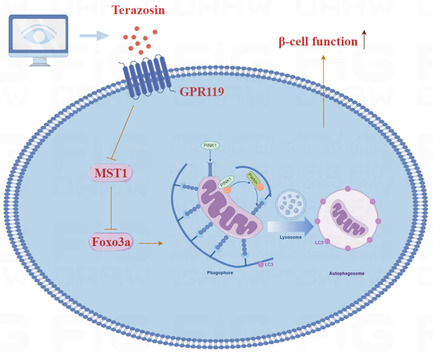

## 1 INTRODUCTION

Obesity is a significant global health challenge linked closely to risks such as type 2 diabetes (T2DM), non‐alcoholic fatty liver disease (NAFLD), and cardiovascular diseases.^1^ These diseases often result from the excess accumulation of fat in the liver, heart, muscles, and kidneys.^2^, ^3^, ^4^, ^5^ As early as 1933, researchers observed and described the phenomenon of fat accumulation in the pancreas as ‘pancreatic lipomatosis’.^6^ Currently, the term non‐alcoholic fatty pancreatic disease (NAFPD) is commonly used to describe such conditions. The defining feature of NAFPD is the excessive accumulation of fat within the pancreas. Unlike NAFLD, fat accumulation in NAFPD typically spares acini and islets but is predominantly located in the pancreatic stroma.^7^ Research reports indicate that NAFPD prevalence in Asian populations ranges from 16% to 35%.^8^, ^9^ A meta‐analysis of 12,675 individuals revealed a combined NAFPD prevalence of 33%, which increases with age.^10^ Various reports have revealed a close association between NAFPD and the occurrence and development of diseases, including NAFLD, T2DM, prediabetes, pancreatitis, pancreatic tumours, and more.^11^, ^12^, ^13^ Currently, treatment methods primarily focus on improving obesity and controlling blood sugar levels. However, the lack of specific targeted drugs poses a challenge to research in this field.

G protein‐coupled receptors (GPCRs) constitute the largest family of membrane proteins in the body, facilitating signal transduction in numerous physiological processes, and rendering them pivotal drug targets. Roughly 30%–40% of FDA‐approved drugs target GPCRs.^14^ G protein‐coupled receptor 119 (GPR119), a member of class A GPCRs, primarily mediates signalling pathways by coupling with the stimulatory G protein (Gs), activating adenylate cyclase, and inducing cyclic adenosine monophosphate (cAMP). Its primary expression occurs in pancreatic β cells and L cells in the gastrointestinal tract. Owing to this distribution, GPR119 activation directly stimulates β cells to secrete insulin, or by inducing the secretion of glucagon‐like peptide‐1 (GLP‐1) in the intestine, thereby reducing blood glucose levels and safeguarding pancreatic β cells.^15^, ^16^ Previous studies have revealed that in the palmitic acid‐induced pancreatic β cell NAFPD model, GPR119 inhibits the Hippo signalling pathway, targeting the key kinase mammalian STE20‐like kinase 1 (MST1) and members of the Forkhead box (Fox) protein family. This inhibition results in the upregulation of pancreatic/duodenal homeobox protein 1 (PDX1), alleviating lipid deposition‐induced β cell apoptosis and functional impairment.^17^ Consequently, the development of GPR119 agonists is crucial to safeguard the functionality of pancreatic β cells.

Due to the remarkable antidiabetic mechanism of GPR119, numerous pharmaceutical companies have developed a range of GPR119 agonists, including PSN 632408, AR231453, MBX‐2982, GSK1292263, and others. Regrettably, the majority of clinical trials involving agonists have concluded in failure,^18^, ^19^ and repeated administration has proven ineffective in reducing average blood glucose levels.^20^, ^21^ This could be attributed to differences in pharmacological species, pharmacokinetics, and compound properties. Consequently, the development path for GPR119 agonists as novel antidiabetic drugs remains challenging. Recently, drug repurposing technology has emerged as a prominent topic in the field of drug development. In the United States, the National Center for Advancing Translational Sciences defines drug repurposing as the investigation of drugs already approved for treating diseases or symptoms to assess their safety and effectiveness for other conditions.^22^ The ultimate goal is to accurately predict clinical applications, enhance health and quality of life, and simultaneously reduce the time and cost of drug development.^23^ Presently, 30% of FDA‐approved drugs have been rediscovered and repurposed.^24^, ^25^ One approach in drug repurposing technology involves using computer technology to virtually screen drugs based on the principles of ligand‐receptor interactions. This, coupled with in vitro and in vivo experiments, aims to uncover new indications for drugs, providing valuable insights for screening GPR119 agonists as novel antidiabetic drugs.

We initially employed Schrödinger Suites drug design software to screen structurally for drugs that may activate GPR119, focusing on first‐line drugs.^26^ Out of 3622 drugs capable of binding to GPR119, our screening of the top 60 drugs based on docking scores revealed terazosin and ZINC‐6601 as clinically employed drugs for treating hypertension; both are α−1 adrenergic receptor blockers. Literature exploration indicates that 50%–80% of T2DM patients also have hypertension. Furthermore, the likelihood of hypertension patients having T2DM is nearly 2.5 times higher than that of normotensive patients.^27^ Reports demonstrate that terazosin exhibits improvement effects on fasting blood glucose and glycated haemoglobin levels in non‐insulin‐dependent diabetes and hypertensive patients.^28^ Another multicenter prospective study revealed that hypertensive patients with impaired glucose tolerance exhibited slight improvement in glucose tolerance and a significant reduction in glycated haemoglobin after 6 months of terazosin treatment. Moreover, there were significant decreases in serum total cholesterol and triglyceride (TG) levels.^29^ Despite clinical studies observing potential improvements in the lipid metabolism of hypertensive patients with terazosin, its precise role and underlying mechanisms remain undisclosed. This study aims to investigate whether terazosin activates GPR119 and enhances pancreatic β‐cell function via the MST1‐Foxo3a signalling pathway, with a specific focus on addressing NAFPD resulting from lipid deposition. The findings would provide insights into the treatment of patients with hypertension complicated with lipid metabolism disorders.

## 2 MATERIALS AND METHODS

### 2.1 Drug repurposing based on the molecular docking

**FIGURE 1:**
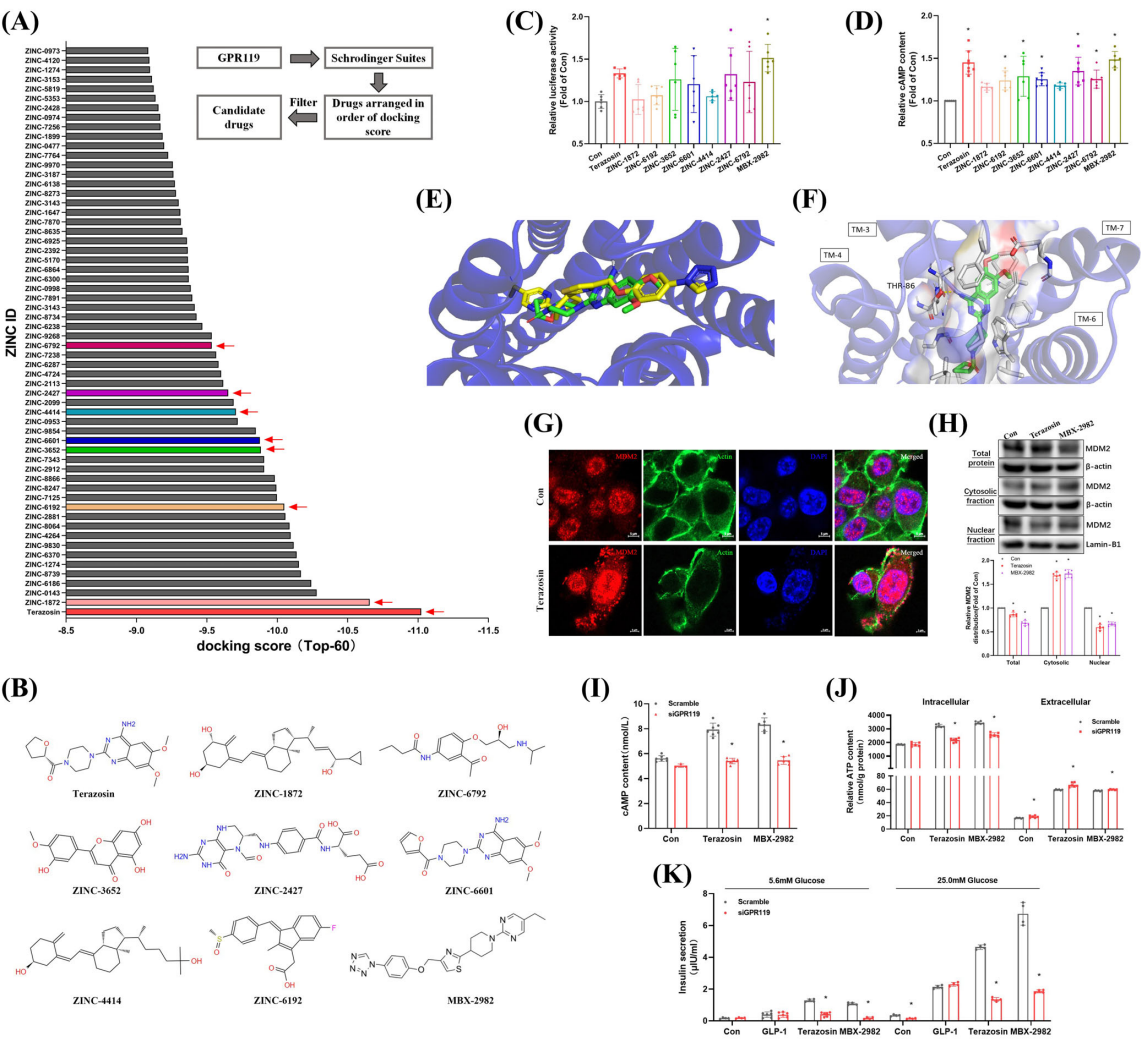
Terazosin selectively activated GPR119, leading to increased insulin secretion in MIN6 cells. (A) Flowchart illustrating the screening of drugs based on structural compatibility with GPR119 and their docking score ranking (Top‐60). (B) Structures of candidate drugs and the positive control MBX‐2982 were depicted. (C) Comparative analysis of CRE promoter transcriptional activity following 24 h treatment of MIN6 cells with candidate drugs (*n* = 6), *p <0.05 versus control group. (D) Comparative assessment of cAMP levels in MIN6 cells after 24 h treatment with candidate drugs (*n* = 6–8), *p <0.05 versus control group. (E), (F) PLIP analysis of terazosin binding to GPR119, with MBX‐2982 as a positive control. (G) Confocal imaging validated the nuclear exclusion of MDM2 in MIN6 cells induced by terazosin. Scale bar = 5 μm. (H) Terazosin decreased the nuclear localization of the MDM2 protein in MIN6 cells, using Lamin‐B1 and *β*‐actin as biomarkers for nuclear and cytosolic fractions (*n* = 5), *p < 0.05 versus control groups of nuclear and cytosolic fractions. (I), (J) Silencing of GPR119 suppressed the terazosin‐induced elevation of cAMP and ATP levels (*n* = 5–6), *p <0.05 versus scramble groups of terazosin or MBX‐2982 induced. (K) Silencing of GPR119 impeded terazosin‐induced glucose‐stimulated insulin secretion, with GLP‐1 and MBX‐2982 serving as positive controls (*n* = 4–6), *p <0.05 versus scramble groups of terazosin or MBX‐2982 induced. Statistical analysis was performed using Student's *t*‐test, Dunnett's *t*‐test, and Mann–Whitney U test.

Receptor Virtual Screening Forms the Basis of GPR119 Ligand Drug Screening. Screening utilizes the Universal Protein (Uniprot) for querying or importing receptor structures (https://www.uniprot.org/uniprotkb/Q7TQP3/entry#structure). The molecular docking technique of the Schrödinger Suites drug design software is utilized. Compounds from a chemical database are automatically matched to the binding site based on the receptor's 3D structure. Scoring functions based on molecular force fields are applied to calculate binding energies for potential binding modes. Ultimately, a ranking list of docking scores is generated. The drug screening process is illustrated in Figure 1A. Using protein‐ligand interaction profiler (PLIP) to analyse the binding conformation and non‐covalent interactions between the drug and GPR119 (https://plip-tool.biotec.tu-dresden.de/plip-web/plip/index).

### 2.2 Cell culture and treatment

The MIN6 mouse pancreatic β‐cell line was cultured in high‐glucose DMEM medium with 10% fetal bovine serum (FBS, Gibco, Carlsbad, CA) under a humidified environment at 5% CO_2_ and 37°C. MIN6 cells were treated with terazosin (20 μmol/L, MCE, HY‐B0371A) and a set of screened drugs (ZINC‐1872 10 μmol/L, ZINC‐6192 5 μmol/L, ZINC‐3652 1 μmol/L, ZINC‐6601 10 μmol/L, ZINC‐4414 1 μmol/L, ZINC‐2427 20 μmol/L, ZINC‐6792 5 μmol/L) for 24 h individually. MBX‐2982 (10 μmol/L, MCE, HY‐15291) served as a positive control. To induce insulin secretion, the cells were transfected with siRNA for 24 h, followed by two washes with preheated Krebs‐Ringer bicarbonate buffer. Subsequently, they were incubated in a medium with 5.6 mmol/L and 25.0 mmol/L glucose for 1 h, respectively. The supernatant was collected for insulin content determination. For the establishment of the NAFPD cell model, 125 μmol/L palmitic acid (PA) was added to the MIN6 cell culture medium for 12 h. Terazosin, if required, was added after 12 h of PA intervention.

### 2.3 Animal treatment

All animal experiments were approved by the Ningxia Medical University Medical Ethical Committee. Male mice aged 6–8 weeks with a C57BL/6 background and GPR119^−/−^ mice were utilized. GPR119^−/−^ mice were generated through CRISPR SpCas9 gene‐editing technology by Weishanglide Biotechnology Co., Ltd., Beijing, China. After 1 week of acclimatization, mice were fed a high‐fat diet (HFD) containing 60% fat (Jiangsu XieTong, XTHF60) for 12 weeks. Daily intraperitoneal injections of terazosin (1.5 mg/kg body weight) or physiological saline were given for 6 weeks. All animals were maintained under controlled conditions (12 h light/dark cycle, temperature approximately 22–25°C), with ad libitum access to food and water.

### 2.4 Plasmid and siRNA transfection

Transfection of Scramble siRNA (20 μmol/L) and mouse GPR119 siRNA (20 nmol/L) into MIN6 cells was conducted for 24 hours using Advanced DNA RNA Transfection Reagent (Zeta life, AD600025). MIN6 cells were transfected individually with Scramble, siMST1, and siFoxo3a, or co‐transfected with Ad‐GFP, Ad‐MST1, and Ad‐Foxo3a according to experimental requirements. Plasmids and siRNA for mouse overexpression were synthesized by Beijing Qiangke Biotechnology Co., Ltd. (China).

### 2.5 Cell viability assay

To evaluate cell proliferation and viability based on diverse experimental needs, we employed the Cell Counting Kit‐8 (CCK‐8, US Everbright, C6005M) and Ki67 staining (Abmart, TW001S). Subsequently, 10 μL of CCK‐8 solution was added to each well in a 96‐well cell culture plate and incubated at 37°C for 1 h. The absorbance value (OD) was then measured at 450 nm using a microplate reader (Thermo Scientific, USA). In the presence or absence of terazosin, cell slides were treated with PA to observe changes in Ki67 fluorescence intensity.

### 2.6 Immunoblotting

Cellular or tissue lysis was conducted with whole protein extraction kit (KeyGEN BioTECH, KGP2100). Fifty micrograms of protein samples were separated by SDS‐PAGE (Epizyme, PG112) and transferred onto a polyvinylidene fluoride membrane. The membrane was incubated overnight at 4°C with the primary antibody, followed by washing with TBST the next day and co‐incubation with horseradish peroxidase‐conjugated secondary antibody for 1 h. Protein observation utilized the highly sensitive chemiluminescent detection kit (US Everbright, S6009L) and the multifunctional molecular imaging system (Azure Biosystems, C600). Image analysis was performed using Image J software with β‐actin as a control. Anti‐GPR119(ab75312) was purchased from Abcam. Anti‐MST1(3682S), anti‐pMST1(49332S), anti‐Foxo3a(12829S), anti‐pFoxo3a(49332S), anti‐PDX1(5679S), anti‐Parkin(2132S), anti‐P62(23214S), anti‐LC3A/B(12741S), anti‐Nkx6.1(54551S), and anti‐caspase‐3(9662S) were purchased from Cell Signalling Technology. Anti‐Bax(50599‐2‐lg) and anti‐PINK1(23274‐1‐AP) were purchased from Proteintech. Anti‐Slc2a2(A12307), anti‐NeuroD1(A1147), anti‐P27(A15632), and anti‐caspase‐1(A0964) were purchased from ABclonal. Anti‐IL‐1β(BS6067) and Anti‐β‐actin (BS6007M) were purchased from Bioworld. Anti‐MDM2(AF0208) was purchased from Affinity. Anti‐Bcl2(40415) was purchased from SAB.

### 2.7 RNA extraction and real‐time quantitative PCR

Cells or tissues were processed for total RNA extraction using the Total RNA Extraction Kit (Omega, R6834). Following the manufacturer's protocol, 1 μg of total RNA was reverse transcribed into cDNA using the Reverse Transcription Kit (Takara, RR036A). The real‐time fluorescence quantitative system (Analytik Jena, qTOWER3G) was employed for the detection of the target gene using RT‐PCR reagents (Takara, RR086A). Relative gene expression analysis was performed using the 2^−ΔΔCT^ method, with *β*‐actin as the internal reference control. The PCR primer sequences are provided in Supplementary Table 1.

### 2.8 Dual‐luciferase reporter gene assay

MIN6 cells were plated in a 12‐well cell culture plate and incubated for 12–24 h prior to transfection. The luciferase reporter genes for Mouse PDX1 and cAMP response element were synthesized by Hunan Fenghui Biotechnology Co., Ltd. (China), with pGL3‐Basic used as a control. Following experimental requirements and manufacturer's instructions, cells were co‐transfected with the mentioned reporter genes (3 μg) and the pRL‐TK Renilla luciferase vector using Advanced DNA RNA transfection reagent (Zeta life, AD600025). The dual luciferase reporter gene assay kit (Beyotime, RG027) was used to quantify the activities of firefly and Renilla luciferases.

### 2.9 cAMP assay

MIN6 cells were seeded in a 6‐well cell culture plate and incubated for 24 h. Subsequently, a 24‐h intervention with terazosin and other drugs was conducted, with MBX‐2982 employed as a positive control. GPR119 siRNA was either transfected or left untransfected during this process. cAMP levels were measured following the manufacturer's instructions, utilizing the cAMP enzyme‐linked immunosorbent assay kit (Nanjing Jiancheng Bioengineering Institute, H164‐1‐2).

### 2.10 ATP measurement

Using the ATP assay kit (Beyotime, S0026), ATP content in cells was measured under conditions of GPR119 siRNA transfection or non‐transfection. Additionally, ATP content in tissues was determined using the same method. Cells or tissues were lysed on ice, and the supernatant was collected by centrifugation at 4°C (12,000 g, 5 min). The ATP detection reagent mixture was added to a 96‐well chemiluminescence plate with a black bottom and cap, followed by luminescence detection using a microplate luminometer (Promega, GloMax navigator). Protein concentration of the samples was determined, and ATP content was standardized to nmol/g protein to minimize errors.

### 2.11 Immunoprecipitation

Following a 24‐h intervention with or without terazosin in MIN6 cells, immunoprecipitation was conducted utilizing the Protein A/G Immunoprecipitation Kit (Epizyme, YJ201). Subsequently, the antigen–antibody‐magnetic bead complexes underwent washing with a dedicated buffer, and protein expression was assessed through immunoblotting experiments. To evaluate the effect of terazosin on protein interactions, MST1 protein levels were normalized, followed by an analysis of changes in Foxo3a protein levels.

### 2.12 Nuclear cytosol extraction

Following the manufacturer's guidelines, we extracted nuclear and cytoplasmic proteins from MIN6 cells, with or without terazosin intervention for 24 h, utilizing the nuclear and cytoplasmic protein extraction kit (BestBio, BB‐3112). Protein expression was assessed via immunoblot experiments, using β‐actin and LaminB1 as controls for cytoplasmic and nuclear proteins, respectively.

### 2.13 Oil Red O staining and TG content measurement

MIN6 cells were cultured in a 12‐well cell culture plate for 24 h, with or without terazosin. Subsequently, the cells were treated with PA (125 μmol/L) for 12 h. After two PBS washes, fixation was carried out using 4% paraformaldehyde for 1 h. Following, Oil Red O staining was conducted utilizing the staining kit (Solarbio, G1261), and cellular observations were made under an inverted microscope. To quantify TG content, cells or tissues subjected to intervention or not were lysed in lysis buffer. TG content was measured using the TG enzymatic assay kit (Applygen, E1013), following the manufacturer's instructions. Concurrently, protein quantification was performed on the samples, and TG content was standardized per milligram of protein concentration (μmol/mg protein) to mitigate errors.

### 2.14 IL‐1β content measurement

MIN6 cells were treated with PA (125 μmol/L) for 12 h, in the presence or absence of terazosin. Mouse IL‐1β levels were measured using the mouse IL‐1β assay kit (MEIMIAN, MM‐0040M1), following the manufacturer's instructions. Cell lysis was achieved through repeated freeze–thaw cycles, followed by centrifugation at 3000 rpm for 20 min to collect the supernatant. Ten microliters of the supernatant, mixed with 40 μL of sample diluent, were added to the enzyme‐labelled plate. Enzyme standard reagent (100 μL per well) was added to the enzyme‐labelled plate. After incubation at 37°C for 1 h, absorbance at 450 nm was measured following wash, coloration, and termination. Standard curves were generated using ELISA calc software, and the actual concentration of IL‐1β in each sample was then calculated.

### 2.15 Immunofluorescent staining

To investigate alterations in the subcellular localization of Foxo3a and MDM2, we prepared cell slides and treated them with terazosin for 24 h. Cells were fixed with 4% paraformaldehyde at room temperature for 1 h and permeabilized with 0.3% Triton X‐100/1% BSA for 20 min. After three washes with PBS, cells were blocked with 5% BSA at room temperature for 30 min. Subsequently, cell slides were incubated overnight at 4°C with anti‐Foxo3a (1:600) and anti‐MDM2 (1:200). Following PBS washing, the cell cytoskeleton was labelled with a microfilament fluorescent probe (1:100, Beyotime, C2201S), and corresponding secondary antibodies (1:100) were added and incubated at room temperature for 1 h. Cell nuclei were stained with DAPI, and imaging was performed using the laser confocal system (Zeiss, LSM800). Depending on experimental needs, cell slides were treated with or without terazosin, and changes in fluorescence intensity of PDX1 and insulin were monitored. Mitochondria were labelled with Mito‐Tracker Red CMXRos (Beyotime, C2205S), LC3B was labelled with pCMV‐GFP‐LC3B plasmid (Beyotime, D2815), and anti‐Parkin antibody (1:50) was utilized to examine alterations in the co‐localization of mitochondria with Parkin protein and autophagosomes. A total of 5 μm sections of mouse pancreatic tissue were prepared from the pancreatic tail, treated with EDTA antigen retrieval solution (Solarbio, C1034) at 95°C for 15 min, and then incubated in 3% H_2_O_2_ for 10 min. Following a 1‐h block with goat serum (ZSGB‐Bio, ZLI‐9022), incubation with the respective primary and secondary antibodies was carried out, and image acquisition was completed.

### 2.16 Mitochondrial damage and autophagy flux analysis

ATP levels (as previously mentioned), intracellular reactive oxygen species (ROS) changes, and mitochondrial membrane potential analysis are employed to evaluate mitochondrial damage and the decline in mitochondrial function. Cells were treated with PA following overnight cell culture, with or without terazosin. According to the manufacturer's instructions, the ROS levels were measured using the ROS detection kit (US Everbright, R6033), and mitochondrial membrane potential was assessed using the JC‐1 mitochondrial membrane potential detection kit (US Everbright, J6004). DCFH‐DA working solution and JC‐1 staining working solution were added separately, and fluorescence changes were observed after incubation at 37°C in the dark. Autophagic flux was estimated by changes in LC3‐II protein expression after treatment with Bafilomycin A1, using the immunoblotting method. Furthermore, autophagic flux was assessed by transfecting cells with the pCDH‐CMV‐mRFP‐GFP‐LC3B‐EF1A‐Puro plasmid (Hunan Fenghui Biotechnology Co., Ltd.) to label LC3B, and observing the fluorescence changes of mRFP and GFP to determine the promotion or inhibition of autophagic flux.

### 2.17 Metabolic phenotype analyses

Mice underwent a glucose tolerance test (GTT) after a 12–16 h fast with a glucose dose of 2 g/kg body weight. Additionally, an insulin tolerance test was carried out on mice following a 4‐h fast with an insulin dose of 0.5 U/kg body weight, with blood glucose levels monitored at various intervals. Prior to euthanasia, serum insulin levels were assessed through the mouse insulin ELISA kit (MEIMIAN, MM‐0579 M1). Simultaneously, a biochemical analyser (Siemens, ADVIA XPT) was employed to quantify blood glucose, lipids (including TG, total cholesterol‐CHOL, high‐density lipoprotein‐HDL, low‐density lipoprotein‐LDL), liver function markers (alanine aminotransferase‐ALT, aspartate aminotransferase‐AST), and renal function indicators (UREA, creatinine‐CREA).

### 2.18 Transmission electron microscopy observation

Collect MIN6 cells or mouse pancreatic tissues treated with terazosin or PA (size<1 mm^3^), fixed in 2.5% glutaraldehyde and 1% osmium tetroxide. After dehydration, embedding, and sectioning, cut ultrathin sections (70 nm) using an ultramicrotome. Subsequently, stained with uranyl acetate and lead citrate for observing cellular ultrastructure via transmission electron microscopy.

### 2.19 Statistical analyses

Data analysis and processing were performed using GraphPad Prism8. Results are expressed as mean ± standard deviation. The significance of difference was determined by Student's *t*‐test (comparison between two groups) and one‐way analysis of variance with Dunnett's *t*‐test correction (comparison among control group and other groups). Parametric tests were conducted on data with normal distribution and homogeneity of variance; otherwise, non‐parametric test (Mann–Whitney U) was applied. The values of *p* <0.05 were considered statistically significant.

## 3 RESULTS

### 3.1 Screening drugs that match GPR119

Employing molecular docking techniques, we conducted virtual screening based on the GPR119 structure. Consequently, 3622 drugs were screened against GPR119 and organized based on their docking scores. Considering pharmacological effects and adverse reactions, we performed additional screening on the top 60 drugs. Drugs without therapeutic value, such as anti‐tumour and hormonal drugs, were excluded, leading to the identification of 8 candidate drugs (Figure 1A). Notably, terazosin and ZINC‐6601 among the selected candidates exhibit antihypertensive properties. Due to the close association between T2DM and hypertension, we selected 8 candidate drugs, including terazosin, to investigate their potential to enhance pancreatic β‐cell function. Furthermore, as a positive control, we chose the established selective GPR119 agonist MBX‐2982 (Figure 1B).

### 3.2 Terazosin selectively activated GPR119, promoting insulin secretion

In MIN6 cells, the effects of the 8 candidate drugs on cell viability varied. Among them, 50 μmol/L ZINC‐6601 and 50 μmol/L ZINC‐4414 significantly reduced cell viability and were therefore excluded from further consideration (Suppl. Figure 1A–C). Terazosin, ZINC‐3652, ZINC‐6601, ZINC‐2427, and ZINC‐6792 activated CRE promoter activity (Figure 1C), leading to an increase in intracellular cAMP levels. The activation effects of ZINC‐1872, ZINC‐6192, and ZINC‐4414 were negligible (Figure 1D). Treating with terazosin, ZINC‐1872, ZINC‐3652, ZINC‐4414, ZINC‐2427, and ZINC‐6792 increased intracellular ATP levels in MIN6 cells (Suppl. Figure 1D), downregulated MST1‐Foxo3a mRNA and protein expression, and upregulated PDX1 expression (Suppl. Figures 1E, F and 2). Terazosin exhibited more comprehensive activation of CRE and cAMP activity, inhibition of MST1‐Foxo3a expression and phosphorylation, and promotion of ATP production compared to other drugs. It also possessed a higher docking score and might be associated with glucose and lipid metabolism. Terazosin had been confirmed as the ultimate selected drug. PLIP analysis reveals that terazosin and MBX‐2982 share a similar binding pocket on GPR119, with overlapping binding conformations (Figure 1E). Terazosin primarily forms hydrogen bonds with threonine‐86 (THR‐86) in the third transmembrane region (TM‐3) of the binding pocket. The docking binding energy of −7.28 kcal/mol indicates a strong binding affinity between terazosin and GPR119 (Figure 1F). In prior studies, we identified a link between GPR119 and MST1, and the extent of pancreatic tissue damage in a high‐fat mouse model correlated with the status of GPR119.^30^ This study aims to explore the mechanism through which terazosin regulates the MST1‐Foxo3a signalling pathway and enhances pancreatic β‐cell function by activating GPR119.

Desensitization is a prevalent phenomenon in most GPCRs. Studies indicated that changes in subcellular localization, particularly the nuclear‐to‐cytoplasmic shift of the ubiquitin ligase double minute 2 homologue (MDM2) protein, served as a predictive biomarker for receptor desensitization.^31^ Terazosin induced MDM2 nuclear exclusion (Figure 1G) and decreased MDM2 protein levels within the MIN6 cell nucleus (Figure 1H). Terazosin elevated intracellular cAMP and ATP levels, demonstrating a specific enhancement in extracellular ATP. GPR119 silencing inhibited the effect of terazosin (Figure 1I, J). Terazosin, serving as a positive control alongside GLP‐1 and MBX‐2982, enhanced insulin secretion in MIN6 cells under both glucose‐stimulated and non‐stimulated conditions. Following GPR119 silencing, terazosin, and MBX‐2982 showed greatly compromised ability in stimulating insulin secretion under high glucose conditions, except for GLP‐1 (Figure 1K).

### 3.3 Terazosin inhibited the MST1‐Foxo3a pathway, enhancing β‐cell function

**FIGURE 2:**
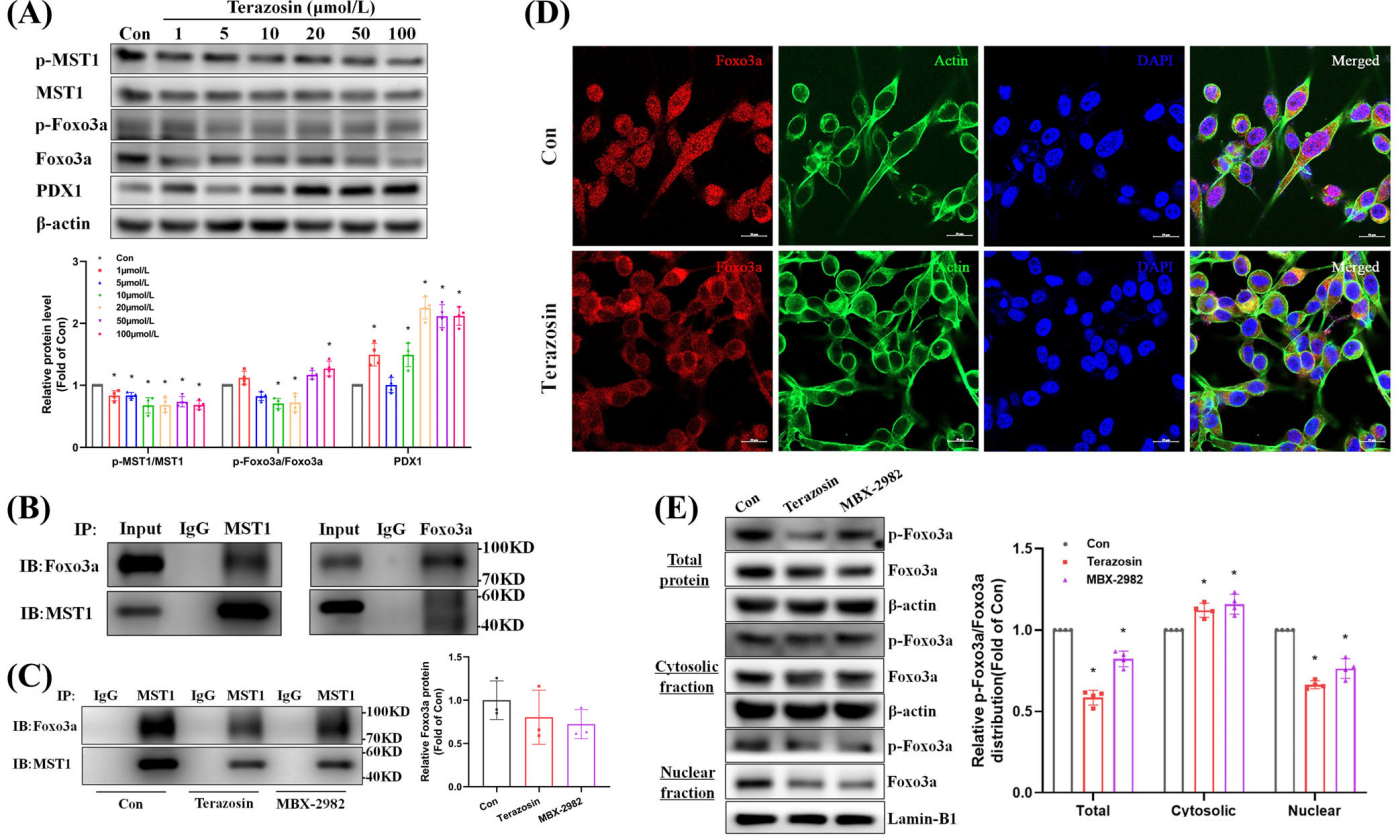
Terazosin inhibited the interaction between MST1 and Foxo3a, leading to the nuclear exclusion of Foxo3a. (A) Terazosin demonstrated dose‐dependent effects on the protein levels of MST1‐Foxo3a and PDX1 in MIN6 cells after a 24‐h treatment with varying terazosin concentrations (*n* = 4), *p <0.05 versus control group. (B), (C) CoIP experiments revealed the interaction between MST1 and Foxo3a in MIN6 cells, with terazosin diminishing this interaction. Quantitative data were displayed on the right (*n* = 4). (D) Confocal imaging confirmed terazosin's role in promoting the nuclear exclusion of Foxo3a in MIN6 cells. Scale bar = 20 μm. (E) Terazosin decreased the nuclear distribution of Foxo3a protein in MIN6 cells, with Lamin‐B1 and *β*‐actin serving as biomarkers for nuclear and cytosolic fractions (*n* = 5), *p <0.05 versus control groups of nuclear and cytosolic fractions. Statistical analysis was performed using Dunnett's *t*‐test.

Terazosin decreased total protein levels and phosphorylation of MST1 and Foxo3a in MIN6 cells, upregulated PDX1 expression, and exhibited a dose‐dependent relationship (Figure 2A). CoIP assays validated the interaction between MST1 and Foxo3a (Figure 2B). Interestingly, terazosin intervention diminished the interaction between MST1 and Foxo3a (Figure 2C). Confirmed by immunofluorescence and immunoblot experiments in MIN6 cells, terazosin decreased total protein levels and nuclear distribution of Foxo3a, with no effect on its cytoplasmic distribution (Figure 2D, E).

**FIGURE 3:**
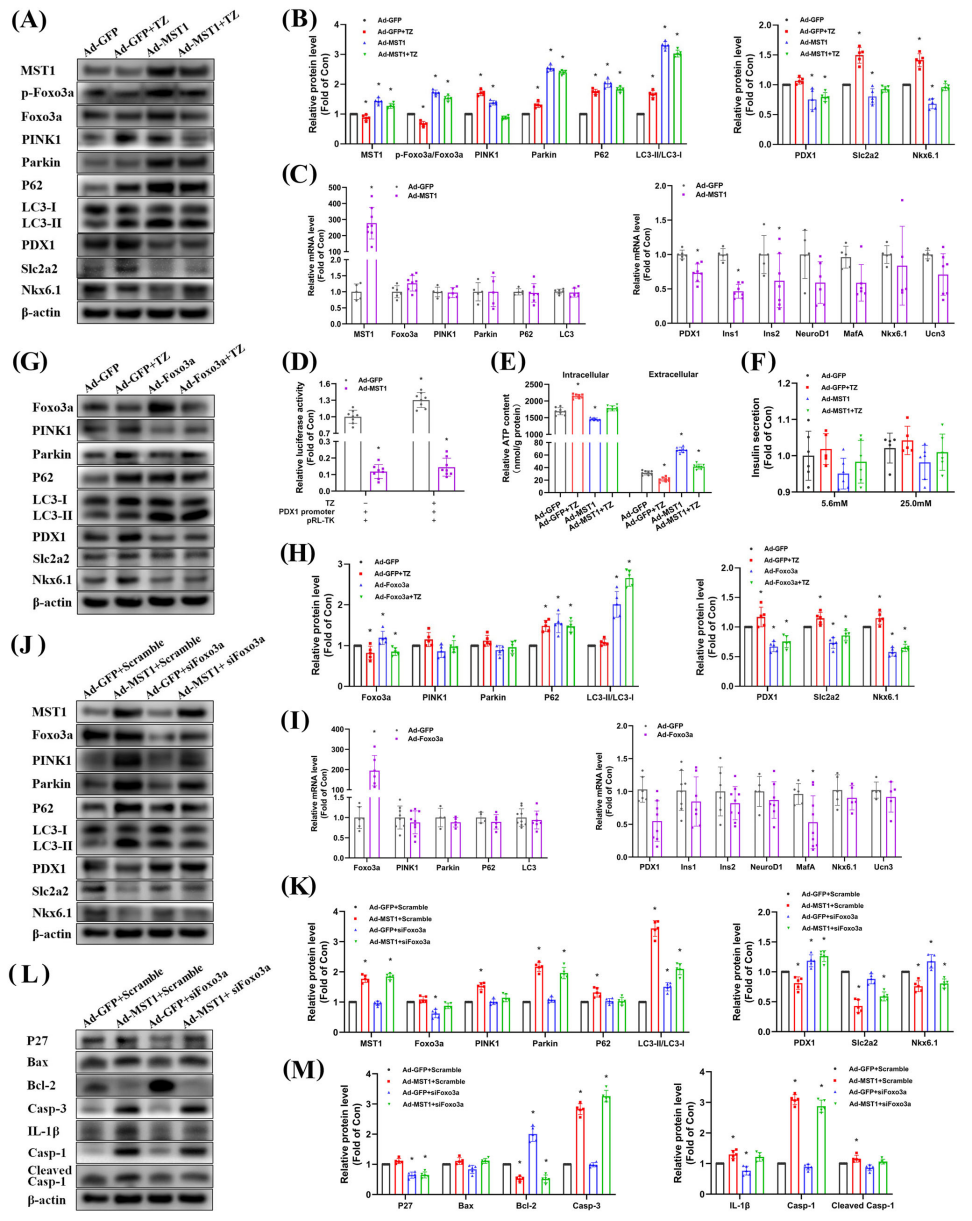
Terazosin inhibited the MST1‐Foxo3a pathway, improving β‐cell function. (A), (B) Terazosin suppressed the upregulation of MST1‐Foxo3a and mitophagy induced by MST1 overexpression in MIN6 cells, accompanied by a reduction in protein levels associated with β‐cell function. Representative gel images were presented in (A) and quantitative data in (B) (*n* = 5), *p <0.05 versus Ad‐GFP groups of various proteins. (C) Real‐time PCR validation of the regulation of gene expression related to MST1‐Foxo3a, mitophagy, and β‐cell function in MIN6 cells overexpressing MST1 (*n* = 5–8), *p <0.05 versus Ad‐GFP groups of various genes. (D) Terazosin prevented the decrease in transcriptional activity of the PDX1 gene promoter induced by MST1 overexpression (*n* = 8), *p <0.05 versus Ad‐GFP group. (E) Terazosin counteracted the reduction in intracellular ATP levels induced by MST1 overexpression (*n* = 8), *p <0.05 versus Ad‐GFP groups of intracellular or extracellular fractions. (F) Terazosin alleviated the reduction in glucose‐stimulated insulin secretion caused by MST1 overexpression (n = 5). (G), (H) Terazosin suppressed the upregulation of Foxo3a and autophagy proteins induced by Foxo3a overexpression in MIN6 cells, concomitant with a reduction in protein levels associated with β‐cell function. Representative gel images were presented in (G) and quantitative data in (H) (*n* = 5), *p <0.05 versus Ad‐GFP groups of various proteins. (I) Real‐time PCR validation of the regulation of gene expression related to Foxo3a, mitophagy, and β‐cell function in MIN6 cells overexpressing Foxo3a (*n* = 5–9), *p <0.05 versus Ad‐GFP groups of various genes. (J), (K) Silencing of Foxo3a inhibited the upregulation of Foxo3a and mitophagy induced by MST1 overexpression in MIN6 cells, accompanied by a decrease in protein levels associated with β‐cell function. Representative gel images were presented in (J) and quantitative data in (K) (*n* = 5), *p <0.05 versus Ad‐GFP + Scramble groups of various proteins. (L), (M) Silencing of Foxo3a suppressed the upregulation of P27, IL‐1β, and Caspase‐1 protein levels induced by MST1 overexpression in MIN6 cells. Representative gel images were presented in (L), and quantitative data in (M) (*n* = 5), *p <0.05 versus Ad‐GFP + Scramble groups of various proteins. TZ, terazosin. Statistical analysis was performed using Student's *t*‐test, Dunnett's *t*‐test, and Mann–Whitney U test.

Our primary goal is to validate the existence of the MST1‐Foxo3a pathway. We observed that overexpressing the MST1 plasmid increased both the mRNA and protein levels of Foxo3a, leading to its phosphorylation (Figure 3A, B). Conversely, silencing MST1 reduced Foxo3a expression (Suppl. Figure 3A, B), suggesting direct regulation by MST1. Terazosin intervention inhibited MST1‐induced upregulation of Foxo3a in MIN6 cells (Figure 3A, B). Subsequently, we investigated various cellular activities to understand how terazosin regulates the MST1‐Foxo3a signalling pathway to enhance β‐cell function. Surprisingly, we identified discrepancies in the regulation of mitophagy mRNA and protein expression due to MST1 overexpression. In contrast to a minor decrease in mRNA, MST1 overexpression markedly elevated protein levels, aligning with terazosin's enhancement of both mRNA and protein expression (Figure 3A–C and Suppl. Figure 3D). Conversely, silencing MST1 concurrently upregulated mitophagy mRNA and protein expression levels (Suppl. Figure 3A, B). Additionally, MST1 overexpression elevated the levels of cell cycle inhibitory, apoptotic, and inflammatory proteins (Suppl. Figure 3E, F), whereas silencing MST1 had the opposite effect (Suppl. Figure 3G). Concerning β‐cell function, MST1 overexpression exhibited an overall downregulation effect (Figure 3A–C), while silencing MST1 upregulated mRNA and protein expression of β‐cell function in MIN6 cells (Suppl. Figure 3A, C). Furthermore, dual‐luciferase reporter gene experiments revealed that both overexpression and silencing of MST1 modulated the promoter activity of the PDX1 gene (Figure 3D and Suppl. Figure 3H), impacting intracellular ATP content and insulin secretion (Figure 3E, F and Suppl. Figure 3I, J). This effect was partially inhibited by terazosin.

Furthermore, we observed regulatory effects of Foxo3a on the cell cycle, apoptosis, inflammation, and β‐cell function, similar to MST1. Foxo3a inhibits mitophagy and the cell cycle while promoting apoptosis and inflammation, resulting in weakened β‐cell function. Terazosin intervention partially reversed Foxo3a's effects (Figure 3G–I and Suppl. Figure 4). We identified Foxo3a's crucial role in the pathway, silencing Foxo3a inhibited MST1 overexpression, increasing mitophagy proteins, upregulating cell cycle inhibitory protein P27, and expressing inflammatory proteins IL‐1β and Caspase‐1. Simultaneously, it prevented the downregulation of proteins related to β‐cell function (Figure 3J–M). Notably, Foxo3a silencing did not inhibit MST1 overexpression‐induced upregulation of apoptosis proteins (Figure 3L, M). In summary, terazosin inhibited the MST1‐Foxo3a signalling pathway, affecting multiple cellular activities, and thereby improving β‐cell function.

### 3.4 Terazosin inhibited the MST1‐Foxo3a pathway, alleviating lipid deposition and mitophagy abnormalities in β‐cell

**FIGURE 4:**
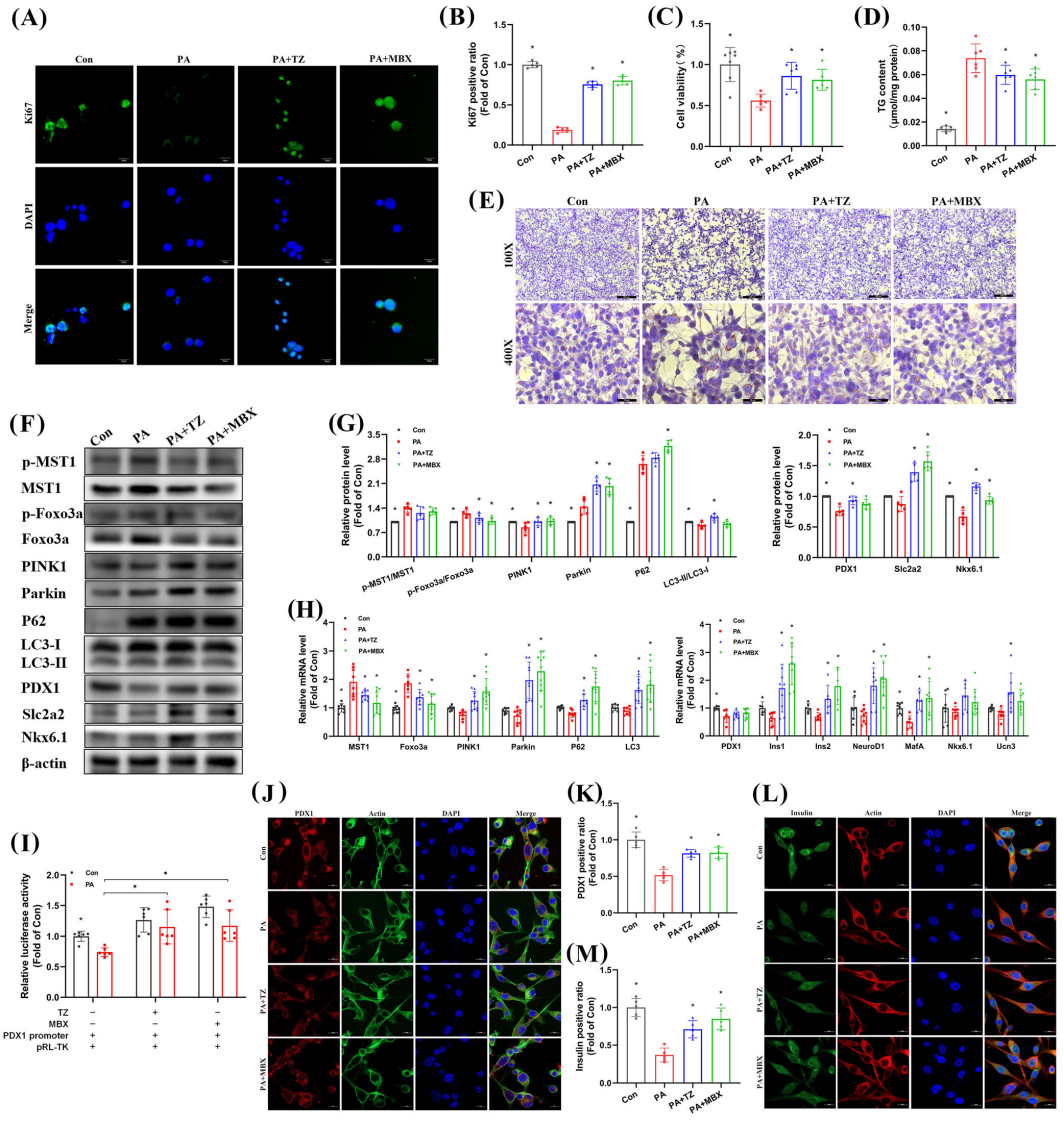
Terazosin inhibited the MST1‐Foxo3a pathway, alleviating lipid deposition and improving β‐cell dysfunction. (A)–(C) Terazosin prevented the decrease in MIN6 cell viability induced by PA. Ki67 protein confocal imaging was shown in (A), Quantitative fluorescence data were presented in (B) (*n* = 5). CCK‐8 data were presented in (C) (*n* = 6), *p <0.05 versus PA group. (D), (E) Terazosin reduced PA‐induced triglyceride (TG) deposition. Quantitative TG data were presented in (D), and Oil Red O staining images were shown in (E). Scale bars: 50 and 200 μm (*n* = 6), *p <0.05 versus PA group. (F), (G) Terazosin inhibited the upregulation of MST1‐Foxo3a and PA‐induced mitophagy in MIN6 cells, and it prevented the downregulation of protein levels associated with β‐cell function. Representative gel images were displayed in (F), and quantitative data in (G) (*n* = 5), *p <0.05 versus PA groups of various proteins. (H) Real‐time PCR confirmed that terazosin inhibited the upregulation of MST1‐Foxo3a and prevented the downregulation of mitophagy and β‐cell function gene expression induced by PA in MIN6 cells (*n* = 6–9), *p <0.05 versus PA groups of various genes. (I) Terazosin prevented the downregulation of PDX1 gene promoter transcriptional activity induced by PA (*n* = 6), *p <0.05 versus PA group. (J), (M) Confocal imaging confirmed that terazosin prevented the downregulation of PDX1 and Insulin protein levels induced by PA in MIN6 cells. Scale bar = 20 μm. Quantitative fluorescence data were presented in (K) and (M) (*n* = 5), *p <0.05 versus PA groups. TZ, terazosin. MBX, MBX‐2982. PA, palmitic acid. Statistical analysis was performed using Dunnett's *t*‐test and Mann–Whitney U test.

Our prior research clarified the MST1 signalling pathway's mechanism in the palmitic acid‐induced NAFLD model.^32^ Nevertheless, the specific mechanism by which terazosin targets MST1 to enhance β‐cell function in NAFPD is still unclear. An in vitro NAFPD model was created by exposing MIN6 cells to 125 μmol/L PA for 12 h. This led to increased levels and heightened phosphorylation of MST1‐Foxo3a proteins, coupled with a decrease in PDX1 protein expression (Suppl. Figure 5A–D). Terazosin intervention improved MIN6 cell proliferation and viability (Figure 4A–C), diminishing PA‐induced TG deposition (Figure 4D, E). Furthermore, terazosin counteracted PA's effects by reducing MST1‐Foxo3a pathway mRNA and protein expression, inhibiting their phosphorylation. This reversal alleviated PA's inhibitory effects on β‐cell function at both mRNA and protein levels, enhancing PDX1 gene promoter activity (Figure 4F–I). Immunofluorescence staining results showed that, compared to PA induction, terazosin intervention in MIN6 cells resulted in increased fluorescence intensity of PDX1 and insulin (Figure 4J–M). It is noteworthy that, similar to MST1 and Foxo3a overexpression, PA‐induced inconsistency in the regulation of mitophagy mRNA and protein expression (Figure 4F–H). Furthermore, terazosin decreased intracellular IL‐1β levels (Suppl. Figure 5E), prevented PA‐induced downregulation of the anti‐apoptotic factor Bcl‐2, and increased the expression of cell cycle inhibitory factors, pro‐apoptotic factors, and inflammatory factors (Suppl. Figure 5F, G). This action thereby enhanced the state of β‐cells in various cellular activities.

**FIGURE 5:**
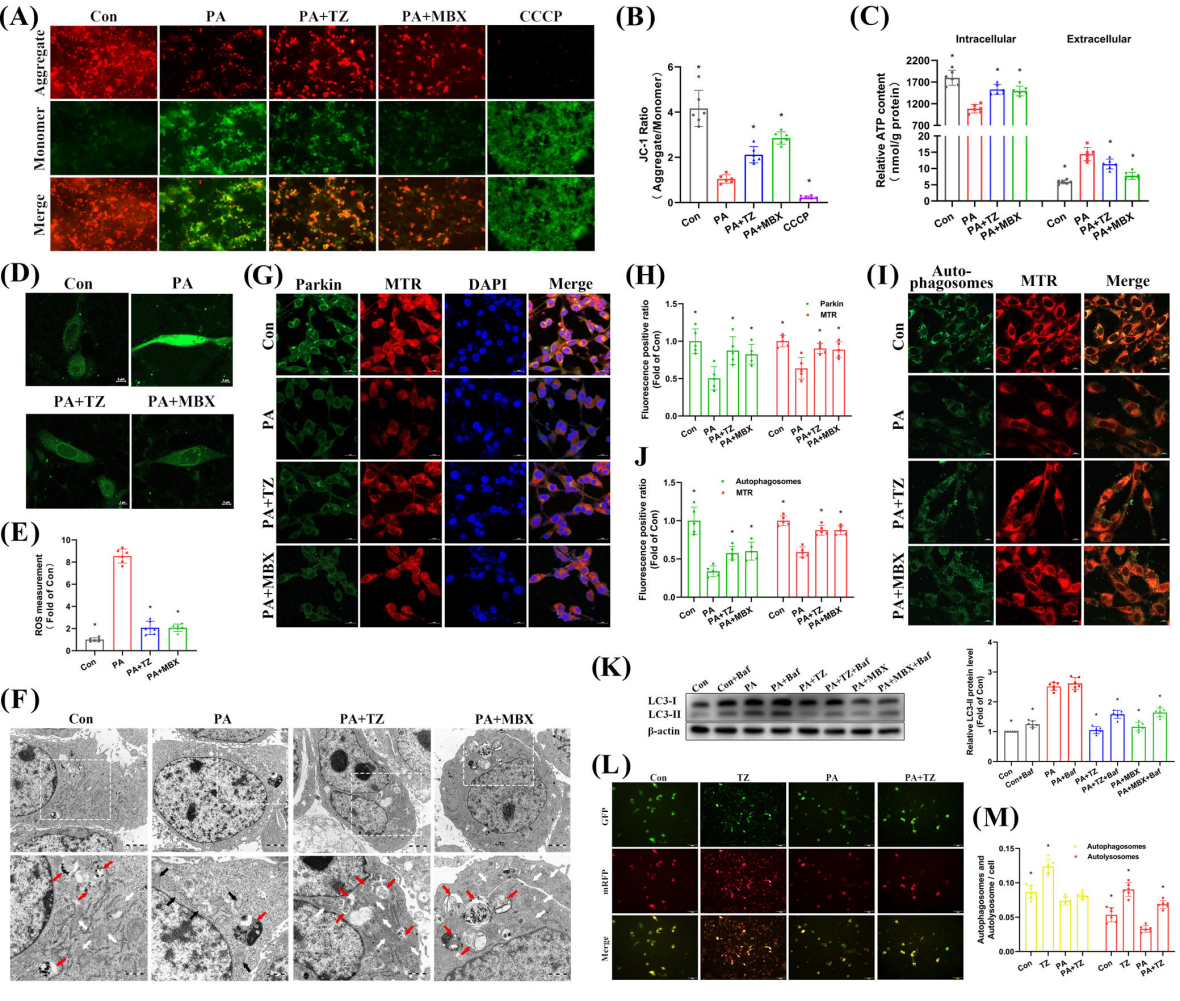
Terazosin prevented mitochondrial damage and abnormalities in autophagic flux induced by PA. (A), (B) Terazosin reversed the decline in mitochondrial membrane potential caused by PA. (A) Shows immunofluorescence staining images: Red fluorescence indicated JC‐1 aggregate under normal membrane potential, and green indicated JC‐1 monomer under membrane potential loss, with CCCP serving as a positive control for membrane potential loss. Scale bar = 100 μm. Quantitative fluorescence data were presented in (B) (*n* = 6), *p <0.05 versus PA group. (C) Terazosin prevented the reduction in ATP content caused by PA in MIN6 cells (*n* = 6), *p <0.05 versus PA groups of intracellular or extracellular fractions. (D), (E) Terazosin decreased ROS production induced by PA. (D) Displays confocal imaging with green fluorescence indicating ROS generation. Scale bar = 5 μm. Quantitative fluorescence data were presented in (E) (*n* = 6), *p <0.05 versus PA group. (F) Transmission electron microscopy images confirmed that terazosin restored the number and structure of mitochondria, leading to an increase in autophagosomes. White arrows indicated normal mitochondria, black arrows indicated swollen mitochondria and red arrows indicated autophagosomes. Scale bar = 1 μm. (G), (H) Confocal imaging confirmed that terazosin enhanced the recruitment of Parkin to mitochondria inhibited by PA. Green fluorescence indicated Parkin protein, red indicated mitochondria, and blue indicated the cell nucleus. Scale bar = 20 μm. Quantitative fluorescence data were presented in (H) (*n* = 5), *p <0.05 versus PA groups. (I), (J) Confocal imaging confirmed that terazosin enhanced the fluorescence of autophagosomes and mitochondria inhibited by PA. Green fluorescence indicated autophagosomes and red indicated mitochondria. Scale bar = 20 μm. Quantitative fluorescence data were presented in (J) (*n* = 5), *p <0.05 versus PA groups. (K) Terazosin enhanced the inhibited autophagic flux caused by PA. Representative gel images were displayed on the left, and quantitative data on the right (*n* = 6), *p <0.05 versus PA group. (L), (M) The tandem fluorescence‐labelled LC3 system confirmed that terazosin effectively restored the inhibited autophagic flux caused by PA. (L) displayed immunofluorescence staining images: Red fluorescence indicated lysosomes, and yellow indicated autophagosomes. Scale bar = 50 μm. Quantitative fluorescence data were presented in (M) (*n* = 6), *p <0.05 versus PA groups. Baf, bafilomycin A1; MTR, mitochondria tracker red; MBX, MBX‐2982; PA, palmitic acid; TZ, terazosin. Statistical analysis was performed using Dunnett's *t*‐test.

Previous research in our group suggested that MST1 regulates lipid accumulation in AML‐12 liver cells via mitophagy.^32^ This study further explored the regulatory role of terazosin in NAFPD‐associated mitophagy. In response to adverse external stimuli, autophagy is initiated by mitochondrial damage, with the reduction in mitochondrial membrane potential being the primary event. We observed that PA caused an elevation in the JC‐1 green fluorescence monomer ratio, indicating a decrease in membrane potential. Simultaneous terazosin intervention led to an increased ratio of red fluorescence aggregates to green fluorescence monomers, effectively restoring the membrane potential (Figure 5A, B). Furthermore, PA decreased intracellular ATP levels (Figure 5C) and induced significant production of mitochondrial ROS (Figure 5D, E). Terazosin intervention effectively inhibited PA's impact on both ATP levels and ROS production. Additionally, transmission electron microscopy was employed to examine the ultrastructure of PA‐stimulated MIN6 cells, with or without terazosin intervention. In the PA‐induced NAFPD model, we observed a decrease in mitochondrial number, loss of normal structure, increased swelling, and mitochondrial damage, accompanied by a reduction in the number of autophagosomes. Terazosin intervention increased mitochondrial numbers, restored normal structure, and enhanced autophagosome abundance, with most being in the late autophagic lysosome stage (Figure 5F). These findings suggested that terazosin effectively repaired mitochondrial damage, restored mitochondrial function, and facilitated the autophagic clearance of damaged mitochondria.

The recruitment of the ubiquitin ligase Parkin and the co‐localization of mitochondria and autophagosomes are crucial steps in the mitophagy process. Terazosin inhibited the decrease in yellow fluorescence intensity induced by PA in the co‐localization of Parkin protein with mitochondria, thereby promoting the recruitment process of Parkin to mitochondria (Figure 5G, H). In the subsequent stage of autophagy, the yellow fluorescence of mitochondria/autophagosomes co‐localization was significantly reduced by PA. Terazosin exhibited the ability to restore this process (Figure 5I, J). Molecular expression at a single time point is insufficient to reflect the entire autophagy process. Dynamic monitoring of changes in autophagic flux can assess the formation, fusion, and degradation of autophagic structures throughout the process. To achieve this, we used Bafilomycin A1, an effective inhibitor of lysosomal fusion and degradation, in conjunction with the tandem fluorescence‐labelled LC3 system. The results showed that terazosin increased the level of LC3‐II protein, further elevated in the presence of BafA1, suggesting that terazosin promoted the autophagic flux process (Suppl. Figure 6A). PA‐induced a significant increase in LC3‐II production, but the presence of BafA1 almost did not further increase its level. This suggests that the inhibition of autophagic flux might be due to defects in lysosomal degradation, and the combined action of terazosin alleviated this inhibition to some extent (Figure 5K). Additionally, terazosin increased the intensity of autophagosome yellow fluorescence and lysosome red fluorescence. It also inhibited the decrease caused by PA in both, thereby to some extent restoring the normal autophagic flux process (Figure 5L, M). These findings suggest that PA‐induced inhibition of autophagic flux might occur at the lysosomal fusion and degradation stages. Terazosin counteracted the effects of PA, promoting the recovery of autophagic flux.

### 3.5 Terazosin treatment improved hyperglycemia, obesity, and pancreatic β‐cell dysfunction in mice with NAFPD

**FIGURE 6:**
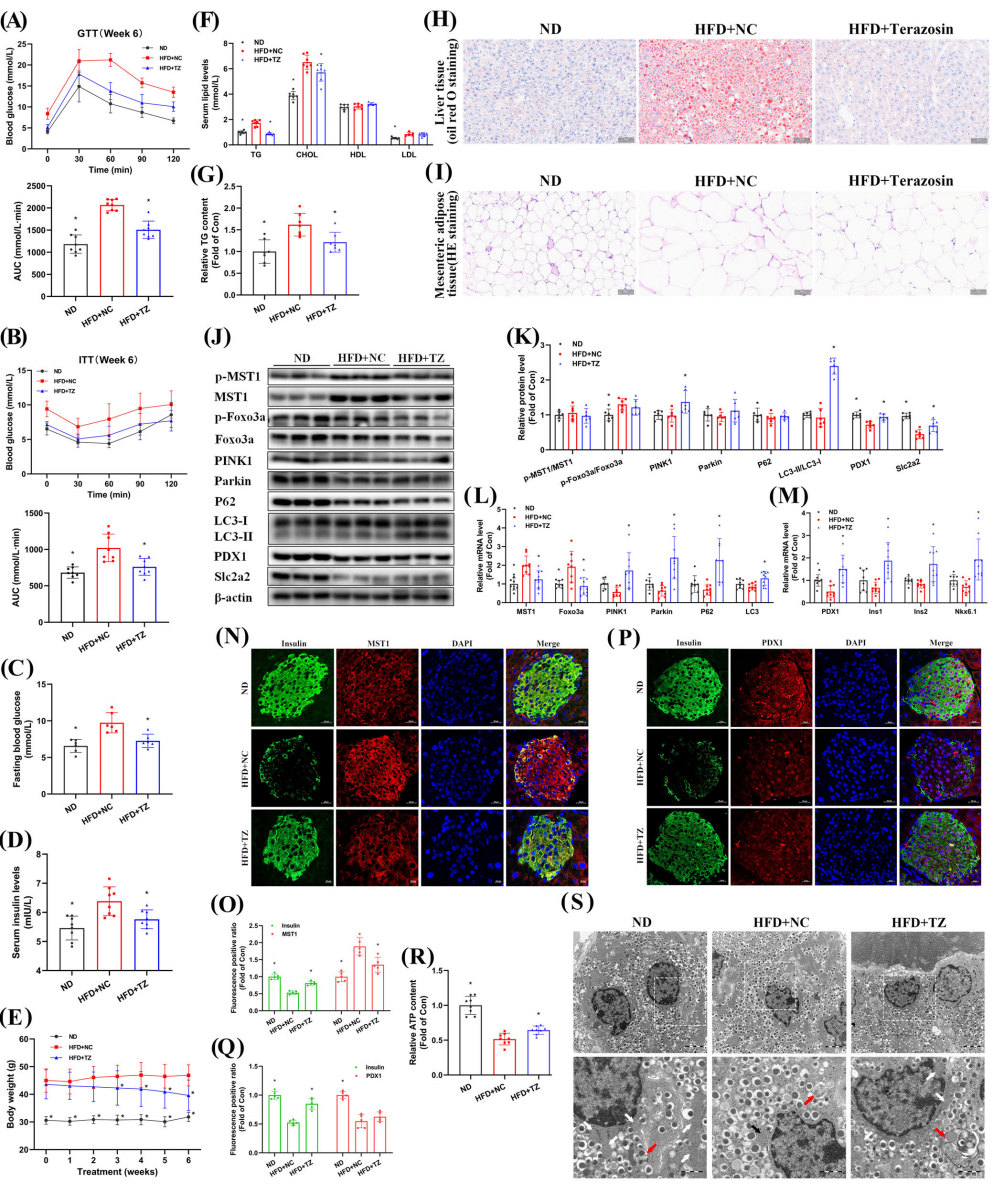
Terazosin treatment ameliorated hyperglycemia, obesity, and pancreatic β‐cell dysfunction in NAFPD mice. (A) Glucose tolerance test (GTT) conducted after 6 weeks of terazosin treatment in NAFPD mice. The upper panel displayed GTT data, while the lower panel showed the corresponding area under the curve (AUC) data (*n* = 8). (B) Insulin tolerance test (ITT) performed after 6 weeks of terazosin treatment in NAFPD mice. The upper panel displayed ITT data, and the lower panel showed AUC data (*n* = 8). (C) A reduction in fasting blood glucose was observed in NAFPD mice following 6 weeks of terazosin treatment (*n* = 6). (D) Terazosin treatment lowered serum insulin levels in NAFPD mice (*n* = 8). (E) Terazosin treatment alleviated obesity in NAFPD mice (*n* = 8). (F) Terazosin treatment reduced serum TG and CHOL levels in NAFPD mice (*n* = 8). (G) Terazosin treatment decreased TG content in the pancreas of NAFPD mice (*n* = 7). (H), (I) Terazosin treatment improved lipid deposition in hepatocytes and mesenteric adipose tissue. Oil Red O staining of hepatocytes was presented in (H), while HE staining of adipose tissue in (I). Scale bar = 50 μm. (J), (K) Terazosin inhibited the upregulation of MST1‐Foxo3a, mitigated mitophagy downregulation, and modulated β‐cell functional protein levels in the pancreas of NAFPD mice. Representative gel images were displayed in (J), while quantitative data in (K) (*n* = 6). (L)–(M) Real‐time PCR verified that terazosin inhibited the upregulation of MST1‐Foxo3a, alleviated mitophagy downregulation, and modulated β‐cell functional gene expression in the pancreas of NAFPD mice (*n* = 7–10). (N)–(Q) Confocal imaging verified that terazosin decreased MST1 protein levels in pancreatic β‐cells of NAFPD mice and elevated levels of PDX1 and Insulin proteins. Scale bar = 20 μm. Quantitative fluorescence data were presented in (O) and (Q) (*n* = 5). (R) Terazosin treatment elevated ATP levels in the pancreas of NAFPD mice (*n* = 8). (S) Transmission electron microscopy images validated that terazosin reinstated mitochondrial number and structure in pancreatic β‐cells of NAFPD mice. White arrows indicated normal mitochondria, black arrows indicated swollen mitochondria and red arrows indicated autophagosomes. Scale bar = 1 μm. ND, normal diet; NC, negative control (mice treated with saline); TZ, terazosin. Statistical analysis was performed using Dunnett's *t*‐test and Mann–Whitney U test, *p <0.05 versus HFD mice treated with saline.

An NAFPD mouse model was created by administering a HFD (Suppl. Figure 6B–D) and treating it with terazosin for 6 weeks to assess its therapeutic effects on NAFPD. Although no significant improvement was observed after 3 weeks of terazosin treatment (Suppl. Figure 6E), a notable enhancement in glucose intolerance was evident after 6 weeks of treatment (Figure 6A). Additionally, terazosin treatment increased insulin sensitivity in NAFPD mice (Figure 6B), leading to a consistent reduction in fasting blood glucose and serum insulin levels (Figure 6C, D). From the third week of terazosin treatment onward, a significant decrease in body weight was observed in NAFPD mice (Figure 6E). Quantitative analysis showed a reduction in TG content in the pancreas of NAFPD mice following terazosin treatment (Figure 6G). Similarly, terazosin consistently lowered serum TG and CHOL levels, with minimal impact on serum HDL and LDL levels (Figure 6F). Furthermore, a significant reduction in serum ALT and AST levels was observed during terazosin treatment (Suppl. Figure 6F). Terazosin treatment had no detrimental effect on the renal function of mice (Suppl. Figure 6G). Staining results of the liver and mesenteric adipose tissue in NAFPD mice showed significant lipid deposition in hepatocytes (Figure 6H) and a marked enlargement in unilocular lipid droplets in adipose tissue (Figure 6I), which was significantly improved after terazosin treatment. Unfortunately, lipid deposition and improvement phenomenon were not significantly observed in islet β cells.

Immunofluorescence staining was used to analyse MST1 expression in mouse pancreatic islets. The results revealed MST1 expression in both pancreatic β cells and other endocrine cells (Suppl. Figure 6H). In NAFPD mouse pancreatic tissue, terazosin treatment inhibited the increase in MST1‐Foxo3a mRNA and protein levels, leading to reduced phosphorylation (Figure 6J–L). Notably, terazosin prevented the decline in mitophagy mRNA and protein levels in the pancreatic tissue of NAFPD mice (Figure 6J–L). Additionally, terazosin treatment inhibited the increase in Bax and Caspase‐3 protein levels, reduced Bcl‐2 protein levels, and concurrently downregulated the expression of P27, IL‐1β, and Caspase‐1 (Suppl. Figure 6I). Terazosin treatment reversed the expression of β‐cell functional related genes at both mRNA and protein levels in NAFPD mouse pancreatic tissue (Figure 6J, K, M). Results of immunofluorescence staining showed that terazosin decreased MST1 fluorescence intensity and effectively prevented the reduction in PDX1 and insulin fluorescence intensity (Figure 6N–Q). Moreover, terazosin treatment elevated the levels of ATP in NAFPD mouse pancreatic tissue (Figure 6R). Transmission electron microscopy revealed a significant reduction in the number of mitochondria in NAFPD mouse pancreatic β cells, accompanied by the loss of normal double‐membrane structure and the presence of autophagic bodies. Terazosin treatment increased the number of mitochondria, restoring the structure to normal, and cells exhibited mitochondrial autophagic bodies (Figure 6S). In summary, these findings suggest that terazosin inhibits the MST1‐Foxo3a signalling pathway, thereby ameliorating hyperglycemia and obesity in NAFPD mice, and facilitating the restoration of pancreatic β‐cell function.

### 3.6 GPR119‐deficiency negated the positive impacts of terazosin treatment on pancreatic β‐cell function in NAFPD mice

**FIGURE 7:**
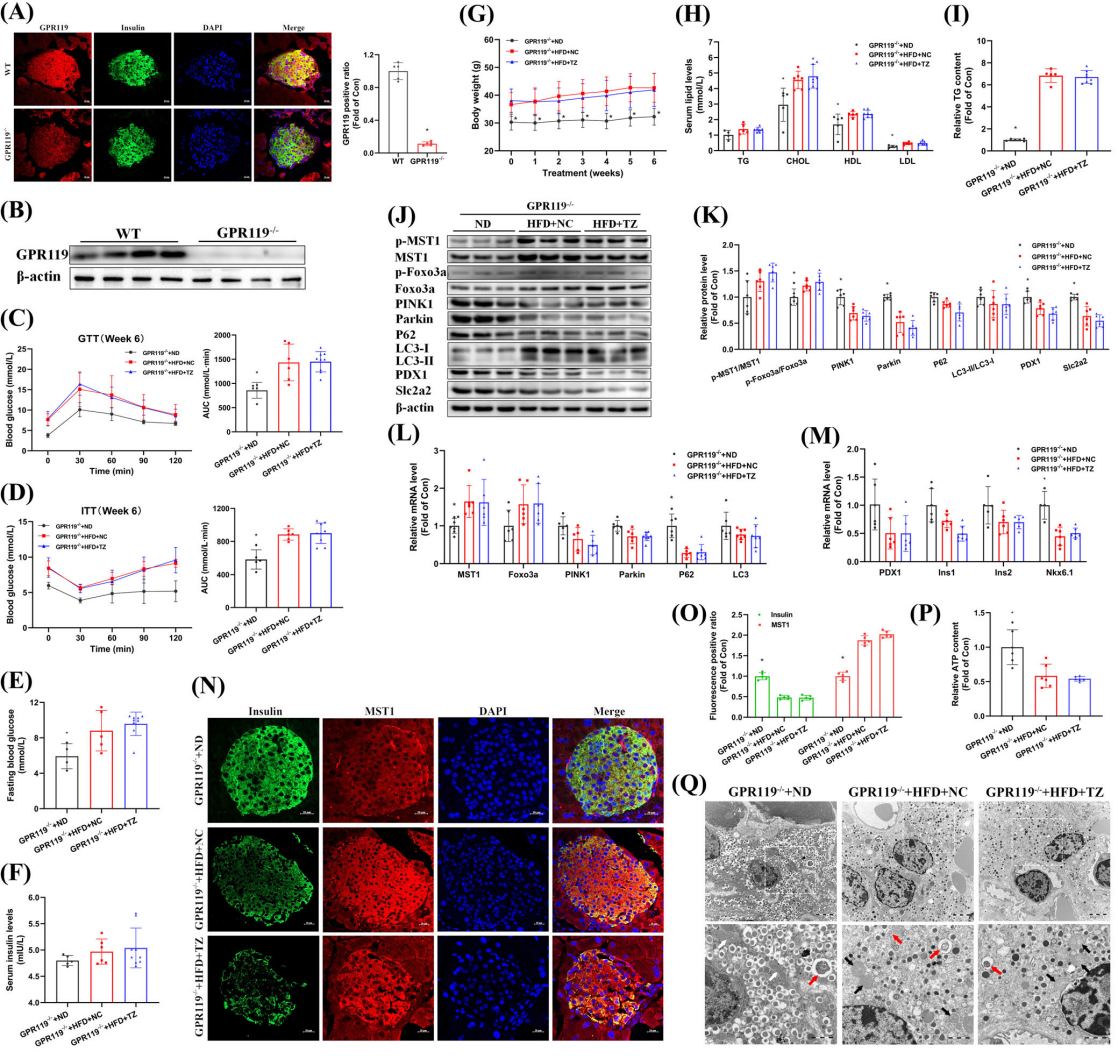
Terazosin treatment failed to ameliorate hyperglycemia, obesity, and pancreatic β‐cell dysfunction in NAFPD mice with GPR119 deficiency. (A), (B) Confocal imaging and immunoblotting validated GPR119 deficiency in mouse islets. (A) displayed confocal images (*n* = 5), Scale bar = 20 μm. Representative gel images were presented in (B). (C) Terazosin treatment failed to ameliorate glucose intolerance in GPR119^−/−^ mice. GTT data were displayed in the left, and AUC data in the right (*n* = 5–8). (D) Terazosin treatment failed to alleviate insulin resistance in GPR119^−/−^ mice. ITT data were displayed in the left, and AUC data in the right (*n* = 5–8). (E) Terazosin treatment failed to reduce fasting blood glucose in GPR119^−/−^ mice (*n* = 5–8). (F) Terazosin treatment failed to decrease serum insulin levels in GPR119^−/−^ mice (*n* = 5–8). (G) Terazosin treatment failed to reduce body weight in GPR119^−/−^ mice (*n* = 5–8). (H) Terazosin treatment failed to decrease serum TG and CHOL levels in GPR119^−/−^ mice (*n* = 5–8). (I) Terazosin failed to reduce TG content in the pancreas of GPR119^−/−^ mice (*n* = 5–8). (J), (K) Terazosin failed to inhibit the upregulation of MST1‐Foxo3a, as well as the downregulation of mitophagy and β‐cell functional protein levels in the pancreas of GPR119^−/−^ mice. Representative gel images were displayed in (J) and quantitative data in (K) (*n* = 6). (L), (M) Real‐time PCR confirmed that terazosin failed to inhibit the upregulation of MST1‐Foxo3a, as well as the downregulation of mitophagy and β‐cell functional gene expression in the pancreas of GPR119^−/−^ mice (*n* = 6). (N), (O) Confocal imaging confirmed that terazosin failed to decrease MST1 protein levels and increase Insulin protein levels in the pancreatic β‐cells of GPR119^−/−^ mice. Scale bar = 20 μm. Quantitative fluorescence data were presented in (O) (*n* = 5). (P) Terazosin treatment failed to increase ATP levels in the pancreas of GPR119^−/−^ mice (*n* = 6). (Q) Transmission electron microscopy images confirmed that terazosin failed to restore the number and structure of mitochondria in the pancreatic β‐cells of GPR119^−/−^ mice. White arrows indicated normal mitochondria, black arrows indicated swollen mitochondria and red arrows indicated autophagosomes. Scale bar = 1 μm. NC, negative control (mice treated with saline); TZ, terazosin. Statistical analysis was performed using Dunnett's *t*‐test and Mann–Whitney U test, *p <0.05 versus GPR119^−/−^ HFD mice treated with saline.

To ascertain the reliance of terazosin's benefits on GPR119 in NAFPD, we created an NAFPD model in GPR119^−/−^ mice subjected to a HFD (Suppl. Figure 7A–C) and treated them with terazosin daily for 6 weeks. Immunofluorescence staining and immunoblotting confirmed GPR119 deficiency in the mouse pancreas (Figure 7A, B). Terazosin treatment did not enhance glucose intolerance and insulin resistance in GPR119^−/−^ mice (Figure 7C, D), lower fasting blood glucose and serum insulin levels (Figure 7E, F), or alleviate weight loss (Figure 7G). Additionally, terazosin treatment did not decrease pancreatic TG content, lower serum TG and CHOL levels in GPR119^−/−^ mice (Figure 7H, I), or serum ALT and AST levels (Suppl. Figure 7D). In the pancreas of GPR119^−/−^ mice, regardless of terazosin treatment, there were no differences in the mRNA and protein levels of MST1‐Foxo3a, and phosphorylation increased similarly (Figure 7J–L). Meanwhile, mitophagy molecules and the anti‐apoptotic factor Bcl‐2 showed no improvement, and cell cycle inhibitory factors, inflammatory factors, and pro‐apoptotic factors remained upregulated (Figure 7J–L and Suppl. Figure 7E, F). Furthermore, terazosin treatment did not elevate the levels of β‐cell functional molecules and immunofluorescence intensity (Figure 7J, K, M–O). Consistently, the levels of ATP in the pancreas remained decreased (Figure 7P). The examination of mitochondria quantity and structure through transmission electron microscopy revealed no improvement, and there was no difference in autophagosomes (Figure 7Q). In conclusion, the absence of GPR119 nullified the positive effects of terazosin treatment on pancreatic β‐cell function in NAFPD mice.

## 4 DISCUSSION

Our earlier studies concentrated on MST1 within the liver, indicating that increased MST1 levels suppressed fat synthesis and enhanced fat oxidation, and proposed diverse materials targeting MST1 for NAFLD treatment, such as microRNA, exosomes, and nanoparticles.^33^, ^34^ Considering the liver and pancreas' location and function correlation, our initial results identified a link between GPR119 and MST1. We verified the connection by observing HFD‐induced pancreatic damage in mice and the concurrent downregulation of GPR119.^21^ Consequently, investigating drugs targeting GPR119 and elucidating their mechanisms in enhancing pancreatic β‐cell function had become the central theme of our research. Terazosin, an antihypertensive drug identified as a GPR119 ligand via molecular docking, was examined for its effects on β‐cell function through in vitro and in vivo experiments, considering its broad activation of GPR119, inhibition of the MST1‐Foxo3a pathway, and potential implications for glucose and lipid metabolism. Nevertheless, it was crucial not to disregard the impact of alternative drugs on β‐cell function, necessitating further exploration. Terazosin, functioning as an α1‐adrenergic receptor blocker, was employed to manage hypertension and relieve urinary symptoms in benign prostatic hyperplasia patients, typically prescribed at a dosage of 10 mg/day. In the cultured mouse pancreatic β‐cell line MIN6, terazosin selectively activated GPR119. It facilitated cell cycle progression, suppressed inflammation and apoptosis, minimized lipid deposition, and enhanced mitochondrial damage repair and autophagic flow, ultimately enhancing β‐cell function. In NAFPD mice, a 1.5 mg/kg terazosin dose markedly ameliorated obesity, hyperglycemia, and insulin sensitivity. Moreover, terazosin treatment in the pancreas of NAFPD mice hindered the MST1‐Foxo3a pathway‐induced downregulation of β‐cell functional mRNA and protein expression. In GPR119‐deficient NAFPD mice, terazosin failed to induce the beneficial effects observed in regular NAFPD mice, including improvements in obesity, hyperglycemia, and insulin sensitivity, as well as the increase of β‐cell functional gene expression in the pancreas. In conclusion, our study demonstrated that terazosin restored normal mitophagy processes and facilitated the recovery of β‐cell function by suppressing the MST1‐Foxo3a signalling pathway. This finding was particularly important for the complex investigation of GPR119 agonist research and development, as well as for treating and restoring β‐cell function in NAFPD.

Terazosin blocks α1‐adrenergic receptors on vascular endothelium, prostate, and bladder smooth muscles. This reduces total peripheral vascular resistance, lowers blood pressure, and relaxes smooth muscles, relieving urethral spasms. Regarding tissue distribution, α1‐adrenergic receptors are mainly found in vascular smooth muscle, myocardium, prostate, and the brain, whereas α2‐adrenergic receptors are expressed in pancreatic tissue.^35^ Therefore, terazosin binds specifically to GPR119 in the pancreas. Currently, our findings validated that terazosin selectively activated GPR119 on the surface of pancreatic β cells, enhancing intracellular CRE promoter activity, increasing cAMP and ATP levels, and regulating the MST1‐Foxo3a signalling pathway, thereby improving β cell function.

In contrast to the previously established positive regulatory role in hepatic lipid metabolism, MST1, a serine/threonine kinase at the core of the HIPPO pathway, has recently been recognized as a pivotal factor in pancreatic β‐cell apoptosis and dysfunction. Identifying and developing inhibitors targeting MST1 have emerged as a novel approach to safeguard β‐cell function.^36^ Drug design methods, relying on structure–activity relationships, identified IHMT‐MST1‐39 and IHMT‐MST1‐58, along with the high‐throughput screening drug Neratinib. These compounds were reported to markedly enhance β‐cell survival, alleviating hyperglycemia, and insulin resistance in diabetic mice.^37^, ^38^, ^39^ The beneficial effects on β‐cell function were also corroborated in this study. However, our emphasis on GPCRs, the primary drug target in drug development, uncovered that among the 3622 drugs screened using molecular docking methods, the top 60, upon re‐screening, included the confirmed activity of terazosin. Of course, this does not suggest that other drugs lack research value; conversely, exploring their mechanisms of action on β‐cell function via GPR119 holds significant research merit.

Mitophagy is a crucial mechanism for intracellular mitochondrial quality control, facilitating the clearance of damaged mitochondria and maintaining mitochondrial homeostasis. Deleting or inhibiting the MST1 gene contributes to restoring mitophagy. Mice with cardiomyocyte‐specific knockout of the MST1 gene show reversed mitophagy, suppressing the apoptotic pathway activated by mitochondria‐promoted apoptotic factors.^40^ In pancreatic cancer cells, MST1 overexpression inhibits mitophagy, promoting the activation of mitochondria‐dependent apoptotic pathways and reducing cell migration.^41^ In this study, we confirmed that terazosin reverses the increased mitochondrial damage induced by MST1 upregulation and validates its positive role in restoring autophagic flux. Consistent with this study's findings, specific knockout of the MST1 gene in diabetic cardiomyopathy mice promotes the autophagic flux process,^40^ as evidenced by increased levels of LC3‐II in the presence of Bafilomycin A1. Considering that autophagic flux involves the formation, fusion, and degradation of autophagic structures, disruptions in these stages can inhibit autophagic flux. Our research reveals an unexpected result: MST1 overexpression suppresses mitophagy gene expression, yet protein levels show an increasing trend. Perhaps a few reported cases can explain this occurrence. In mouse embryonic fibroblasts and myocytes, MST1 loss results in reduced autophagosome‐lysosome colocalization, significant accumulation of autophagic structures, high levels of LC3‐II and P62, and impaired autophagic flux.^42^ Similar to the varied regulatory roles of MST1 in the liver and pancreas, we suspect that pancreatic MST1 may have a regulatory role contrary to previous reports. However, further research is required to confirm this. Furthermore, unlike its regulation of other cellular activities, MST1 overexpression is not affected in its activation of cell apoptosis by Foxo3a silence. It has been reported that MST1 is a direct target and activator of caspase. While initiating the cascade reaction of cell apoptosis, its activity is enhanced by caspase, promoting the apoptotic response circuit.^43^, ^44^ The bidirectional interaction between MST1 and caspase mechanisms determines that their regulation of cell apoptosis relies not only on the status of downstream targets but also on mutual influence.

The forkhead box protein O3a (Foxo3a) is a research hotspot in tumour and drug resistance fields. Although there are few reports on its involvement in metabolic diseases, it unveils a crucial potential role. During streptozotocin (STZ)‐induced diabetes stress in pancreatic β‐cells, Foxo3a inhibits Parkin‐mediated mitochondrial recruitment and mitophagy, thereby impacting the growth of pancreatic β‐cells and insulin secretion.^45^ In peritoneal macrophages of diabetic mice, Foxo3a, through acetylation, reduces PINK1‐dependent mitophagy and inflammasome activation.^46^ Additionally, the association between MST1 and Foxo3a has been investigated and confirmed. The reduction in MST1 activity inhibiting Foxo3a activation might be linked to abnormal neural activity patterns and memory impairment.^47^ Another study reported that MST1 activation results in phosphorylation and nuclear accumulation of Foxo3a, thereby inhibiting tumour cell migration.^48^ In this study, we elucidated the direct regulation of Foxo3a by MST1, enhancing our comprehension of its involvement in diseases related to β‐cell dysfunction. Terazosin inhibiting the MST1‐Foxo3a signalling pathway through GPR119 is a crucial novel mechanism that elucidates the suppression of mitophagy and compromised β‐cell function in NAFPD conditions.

Given the growing correlation between NAFPD and MetS, a disease resulting from the unhealthy lifestyles and dietary habits of contemporary individuals, there is a proposal to incorporate NAFPD as a pancreatic manifestation of MetS in its definition.^49^, ^50^ As obesity progresses and becomes more prevalent, the incidence of NAFPD rises.^51^, ^52^ Elevated pancreatic fat content contributes to impaired glucose tolerance and insulin resistance in the ‘prediabetes’ stage.^53^ Given the direct association of NAFPD with excessive fat intake, an experimental NAFPD model in mice can be induced using a HFD. This leads to ‘prediabetes’ symptoms arising from excessive pancreatic fat accumulation within 12–18 weeks, aligning with the results presented in this study.^54^, ^55^ Notably, reports suggest ‘rediabetes’ may be a risk factor for NAFPD, indicating a potentially vicious cycle.^56^ Conversely, the correlation between hypertension and NAFPD is relatively weak.^57^ Concerning pancreatic fat accumulation, it is crucial to note: firstly, given the physiological presence of a small amount of fat, describing excessive accumulation as ‘in situ’ fat is more appropriate for NAFPD than the ‘ectopic’ fat seen in NAFLD. Second, although the pancreas and liver originate from the same endoderm embryologically,^58^ and their etiologies, correlations, and relationships with other diseases exhibit similarities,^7^ there is a significant difference in fat deposition between the pancreas and liver, a difference also commonly found in various tissues and organs. Fat deposition in the liver is concentrated within hepatocytes, resulting in fat vacuoles of varying sizes. In contrast, fat deposition in the pancreas, or rather fat infiltration, tends to occur primarily within the pancreatic stroma,^7^ rather than being concentrated in the islet cells and acini. Correspondingly, in our study, HFD mice at 12 weeks exhibited significant increases in body weight and blood lipid levels, with substantial fat deposition observed in the liver and adipose tissue, reflecting the model's success and the widespread presence of ectopic fat deposition. However, we did not observe lipid accumulation within the islet cells. This suggests that at the 12‐week time point induced by HFD, lipid deposition in the pancreas is less pronounced than in the liver and adipose tissue, possibly requiring extended time points and further validation to observe more typical phenomena.

Regarding the pathogenic mechanism, our results confirmed that NAFPD lipotoxicity promoted cell inflammation and apoptosis, inhibited the cell cycle, damaged mitochondria, and impaired the autophagic process, weakening β‐cell function across various cellular activities. Presently, most reports support the speculation that fat‐induced inflammatory states lead to impaired β‐cell function. In PA‐induced MIN6 cells, the expression of cytokines interleukin‐1 and interleukin‐6 is upregulated.^59^ In HFD mice, the proportion of perivascular mononuclear inflammatory cell aggregates around the islets significantly increases, and the area of Ly6C‐positive activated macrophage markers within the islets also markedly increases.^54^ Reports also indicate signs of dedifferentiation and loss of function in pancreatic islet β‐cells in HFD mice.^60^ Excessive saturated fatty acids may induce differentiation of pancreatic ductal cells into fat cells, leading to increased fat accumulation.^61^ Currently, foundational reports on the pathogenic mechanism of NAFPD are lacking. This study provided evidence for exploring the mechanism from multiple perspectives. As confirmed in animal models, diet plays a significant role in the pathogenesis of NAFPD. Therefore, the treatment of NAFPD should prioritize influencing factors such as diet control and weight loss,^12^ especially in the absence of large randomized clinical trials evaluating NAFPD medications. In small‐scale animal and clinical trials, the GLP‐1 analog liraglutide has been proven to alleviate the severity of NAFPD.^62^ Unfortunately, exenatide's effect on reducing ectopic fat storage is limited to the epicardium and liver and is ineffective for NAFPD.^63^ In this study, terazosin demonstrated beneficial effects on hyperglycemia, obesity, and NAFPD by activating GPR119 rather than acting as a GLP‐1R or analog. Considering that both NAFPD and hypertension are considered symptoms within the MetS category, our experimental results strongly suggested that terazosin could be prioritized as a therapeutic drug for NAFPD patients with concurrent hypertension or those in the ‘pre‐diabetic’ stage.

Our study unveiled a novel mechanism governing aberrant β‐cell function in NAFPD conditions. The MST1‐Foxo3a signalling pathway emerged as a central player, driving mitochondrial damage, and suppressing autophagic flux. Significantly, this represents the inaugural successful drug repurposing strategy to screen GPR119 agonists, exemplified by terazosin, and assess their influence on β‐cell function in NAFPD. Furthermore, the enhancement of mitophagy through terazosin‐induced activation of GPR119 is documented for the first time. Nevertheless, several issues remain unresolved. Additional experiments are required to elucidate the regulatory mechanisms of GPR119 activation on MST1 and its phosphorylation, encompassing the involvement and regulation of cAMP and PKA in this process. Moreover, a more comprehensive understanding of the adverse effects of mitochondrial damage and autophagy inhibition on β‐cell function, along with their roles in the pathogenesis of NAFPD, is still required. Hence, forthcoming research should concentrate on enhancing the integrity of the GPR119‐MST1‐Foxo3a signalling pathway and refining the analysis of autophagic flux alterations at different stages in NAFPD conditions, including their potential mechanisms. Furthermore, terazosin exhibits common side effects like weakness, palpitations, nausea, peripheral edema, dizziness, and drowsiness. Despite confirming the absence of adverse effects on renal function, its inherent side effects should be taken into account in researching its potential clinical applications for treating NAFPD and hypertension patients. This aspect is also a crucial future avenue for our study.

## AUTHOR CONTRIBUTIONS

**Chenglei Zhang**: Conceptualization, Validation, Formal analysis, Methodology, Data curation, Software, Visualization, Writing‐original draft, Writing‐ review & editing. **Jiarui Li**: Conceptualization, Validation, Methodology, Data curation, Software, Visualization. **Lijuan Wang**: Conceptualization, Methodology. **Jie Ma**: Methodology, Data curation. **Xin Li**: Software, Data curation. **Yuanyuan Wu**: Methodology, Data curation. **Yanru Ren**: Methodology, Data curation. **Yanhui Yang**: Investigation, Supervision. **Hui Song**: Investigation, Supervision. **Jianning Li**: Conceptualization, Methodology, Supervision, Project administration, Funding acquisition. **Yi Yang**: Conceptualization, Supervision, Resources, Writing‐review & editing, Project administration, Funding acquisition.

## FUNDING INFORMATION

This study was supported by grants from the National Natural Science Foundation of China (82160171, NSFC8246031120), Natural Science Foundation of Ningxia Province (2023AAC02038), and Key research and development program of Ningxia Province (2023BEG02020).

## CONFLICT OF INTEREST STATEMENT

The authors have no conflicts of interest to declare.

## Supporting information


**Suppl Figure 1.** Comparative analysis of the effects of candidate drugs. (A)–(C) Comparison analysis of cell viability following 24 h treatment of MIN6 cells with candidate drugs at varying concentrations. (D) Comparison assessment of intracellular and extracellular ATP levels in MIN6 cells after 24 h treatment with candidate drugs. (E), (F) Immunoblotting and real‐time PCR validation of the regulation of MST1‐Foxo3a and PDX1 expression by candidate drugs.
**Suppl Figure 2**. Comparative analysis was conducted to assess the impact of candidate drugs at varying concentrations, alongside the positive control MBX‐2982. After 24 h of treatment with alternative drugs at various concentrations, MST1‐Foxo3a protein expression was downregulated, while PDX1 protein expression was upregulated in MIN6 cells. Representative gel images were presented above, and quantitative data were presented below. *N* = 4. *p <0.05 versus control group.
**Suppl Figure 3**. MST1 expression negatively correlated with β‐cell function. (A) MST1 silencing enhanced mitochondrial autophagy and increased protein levels associated with β‐cell function. Representative gel images were displayed on the left, while quantitative data on the right. (B), (C) MST1 silencing modulated Foxo3a, mitochondrial autophagy, and gene expression associated with β‐cell function, as validated by real‐time PCR. (D) Terazosin upregulated mRNA expression of mitochondrial autophagy. (E), (F) Terazosin inhibited the upregulation of cell cycle inhibition proteins, apoptosis proteins, and inflammatory protein levels induced by MST1 overexpression in MIN6 cells. Representative gel images were shown in (E), and quantitative data in (F). (G) MST1 silencing regulated the expression of cell cycle, apoptosis, and inflammatory proteins. Representative gel images were shown on the left and quantitative data on the right. (H) MST1 silencing upregulated the transcriptional activity of the PDX1 gene promoter. (I) MST1 silencing upregulated intracellular ATP levels. (J) MST1 silencing increased glucose‐stimulated insulin secretion. *N* = 5–8. *p <0.05 versus Ad‐GFP or scramble groups. TZ, terazosin.
**Suppl Figure 4**. Terazosin inhibited the negative regulation of Foxo3a on β‐cell function. (A) Foxo3a silencing upregulated mitochondrial autophagy and protein levels related to β‐cell function. Representative gel images were displayed on the left and quantitative data on the right. (B), (C) Foxo3a silencing regulated real‐time PCR validation of mitochondrial autophagy and gene expression related to β‐cell function. (D), (E) Terazosin inhibited the upregulation of cell cycle inhibitory proteins, apoptosis proteins, and inflammatory protein levels induced by Foxo3a overexpression in MIN6 cells. Representative gel images were shown in (D), and quantitative data in (E). (F) Terazosin prevented the decrease in transcriptional activity of the PDX1 gene promoter induced by Foxo3a overexpression. (G) Terazosin counteracted the reduction in intracellular ATP levels induced by Foxo3a overexpression. (H) Terazosin alleviated the reduction in glucose‐stimulated insulin secretion caused by Foxo3a overexpression. (I) Foxo3a silencing regulated cell cycle, apoptosis, and inflammatory protein expression. Representative gel images were displayed on the left and quantitative data on the right. (J) Foxo3a silencing upregulated PDX1 gene promoter transcriptional activity. (K) Foxo3a silencing upregulated intracellular ATP levels. (L) Foxo3a silencing increased glucose‐stimulated insulin secretion. *N* = 5–8. *p <0.05 versus Ad‐GFP or scramble groups. TZ, terazosin.
**Suppl Figure 5**. Terazosin inhibited PA‐induced alterations in the cell cycle, apoptosis, and inflammatory protein levels in MIN6 cells. (A) Dose‐effect of PA on the expression of MST1‐Foxo3a and PDX1 proteins in MIN6 cells. Representative gel images were shown on the left and quantitative data on the right. (B) Dose‐effect of PA on the activity of MIN6 cells. (C) Time‐dependent effect of PA (125 μmol/L) on MST1‐Foxo3a and PDX1 proteins expression in MIN6 cells. Representative gel images were shown on the left and quantitative data on the right. (D) Time‐dependent effect of PA (125 μmol/L) on the activity of MIN6 cells. (E) Terazosin inhibited the upregulation of IL‐1β levels by PA in MIN6 cells. (F), (G) Terazosin inhibited the PA‐induced upregulation of cell cycle inhibitory proteins, apoptosis proteins, inflammatory proteins, and mRNA levels in MIN6 cells. Representative gel images and quantitative data were shown in (F). Real‐time PCR validation data were shown in (G). *N* = 5–8. *p <0.05 versus cells treated with PA. MBX, MBX‐2982; TZ, terazosin.
**Suppl Figure 6**. Terazosin treatment ameliorated hyperglycemia, obesity, and pancreatic β‐cell dysfunction in NAFPD mice. (A) Terazosin promoted the autophagic flux. Representative gel images were shown in the upper panel and quantitative data in the lower panel. (B) High‐fat diet established the weight curve in the NAFPD mouse model. Weight data were presented in the upper panel, and AUC data in the lower panel. (C) GTT conducted in NAFPD mice before terazosin treatment. GTT data were shown in the upper panel, and AUC data in the lower panel. (D) ITT performed in NAFPD mice before terazosin treatment. ITT data were shown in the upper panel, and AUC data in the lower panel. (E) Despite 3 weeks of terazosin treatment, there was no significant improvement in glucose intolerance in NAFPD mice. GTT data were shown in the upper panel, and AUC data in the lower panel. (F) Terazosin treatment reduced serum ALT and AST levels in NAFPD mice. (G) Terazosin treatment had no impact on kidney function in NAFPD mice. (H) Confocal imaging verified the expression of MST1 in mouse pancreatic endocrine cells. Scale bar = 20 μm. (I) Terazosin inhibited the upregulation of cell cycle inhibition proteins, apoptosis proteins, and inflammatory protein levels in the pancreas of NAFPD mice. Representative gel images were shown in the upper panel and quantitative data in the lower panel. *N* = 6–9. *p < .05 versus HFD mice treated with saline. NC, negative control (mice treated with saline); TZ, terazosin.
**Suppl Figure 7**. Terazosin treatment failed to ameliorate hyperglycemia, obesity, and pancreatic β‐cell dysfunction in NAFPD mice with GPR119 deficiency. (A) High‐fat diet established the weight curve in the NAFPD mouse model with GPR119 deficiency. Weight data were shown in the left panel, and AUC data in the right panel. (B) GTT conducted in GPR119^−/−^ mice before terazosin treatment. GTT data were shown in the left panel, and AUC data in the right panel. (C) ITT performed in GPR119^−/−^ mice before terazosin treatment. ITT data were shown in the left panel, and AUC data in the right panel. (D) Terazosin treatment failed to decrease serum ALT and AST levels in GPR119^−/−^ mice. (E), (F) Terazosin failed to inhibit the upregulation of cell cycle inhibitory proteins, apoptosis proteins, and inflammatory protein levels in the pancreas of GPR119^−/−^ mice. Representative gel images were shown in (E) and quantitative data in (F). *N* = 6–9. *p <0.05 versus HFD GPR119^−/−^ mice treated with saline. NC, negative control (mice treated with saline); TZ, terazosin.
**Supplement Table 1**: Primer sequences used in qRT‐PCR analysis.

## Data Availability

The data that support the findings of this study are available from the corresponding author upon reasonable request.
