# Supplementary material for: Terazosin, a repurposed GPR119 agonist, ameliorates mitophagy and β‐cell function in NAFPD by inhibiting MST1‐Foxo3a signalling pathway
Source: Cell Prolif. 2024 Oct 16;58(3):e13764. doi: 10.1111/cpr.13764 (PMC11882769; doi:10.1111/cpr.13764)
Supplement: Supplementary file 1 — Suppl Figure 1. Comparative analysis of the effects of candidate drugs. (A)–(C) Comparison analysis of cell viability following 24 h treatment of MIN6 cells with candidate drugs at varying concentrations. (D) Comparison assessment of intracellular and extracellular ATP levels in MIN6 cells after 24 h treatment with candidate drugs. (E), (F) Immunoblotting and real‐time PCR validation of the regulation of MST1‐Foxo3a and PDX1 expression by candidate drugs. Suppl Figure 2. Comparative analysis was conducted to assess the impact of candidate drugs at varying concentrations, alongside the positive control MBX‐2982. After 24 h of treatment with alternative drugs at various concentrations, MST1‐Foxo3a protein expression was downregulated, while PDX1 protein expression was upregulated in MIN6 cells. Representative gel images were presented above, and quantitative data were presented below. N = 4. *p <0.05 versus control group. Suppl Figure 3. MST1 expression negatively correlated with β‐cell function. (A) MST1 silencing enhanced mitochondrial autophagy and increased protein levels associated with β‐cell function. Representative gel images were displayed on the left, while quantitative data on the right. (B), (C) MST1 silencing modulated Foxo3a, mitochondrial autophagy, and gene expression associated with β‐cell function, as validated by real‐time PCR. (D) Terazosin upregulated mRNA expression of mitochondrial autophagy. (E), (F) Terazosin inhibited the upregulation of cell cycle inhibition proteins, apoptosis proteins, and inflammatory protein levels induced by MST1 overexpression in MIN6 cells. Representative gel images were shown in (E), and quantitative data in (F). (G) MST1 silencing regulated the expression of cell cycle, apoptosis, and inflammatory proteins. Representative gel images were shown on the left and quantitative data on the right. (H) MST1 silencing upregulated the transcriptional activity of the PDX1 gene promoter. (I) MST1 silencing upregulated intracell [file CPR-58-e13764-s001.docx]

**Suppl Fig.1**


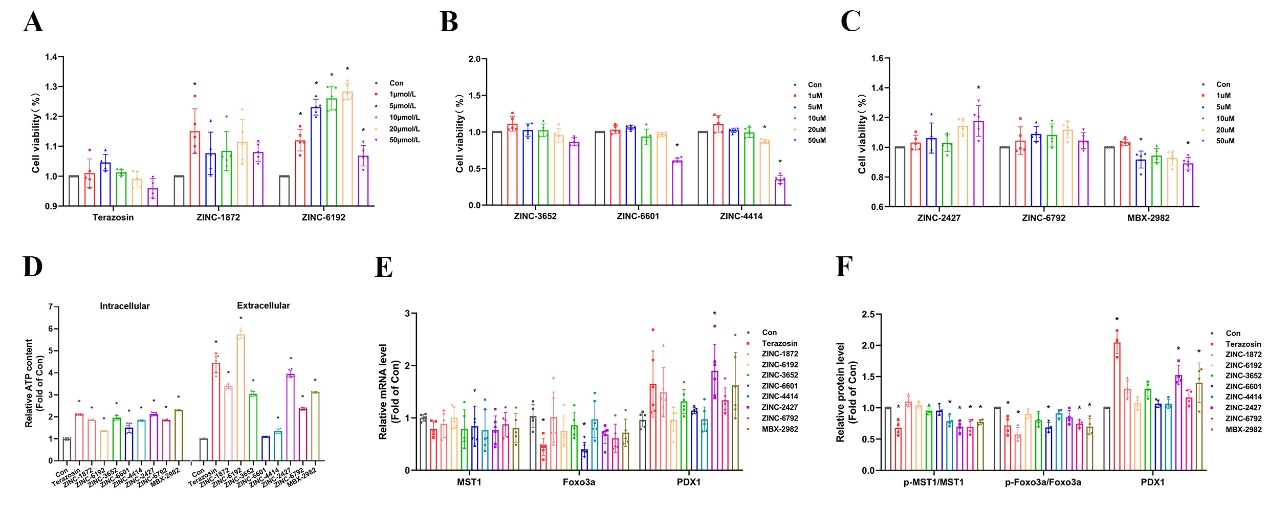


**Suppl Fig.2**


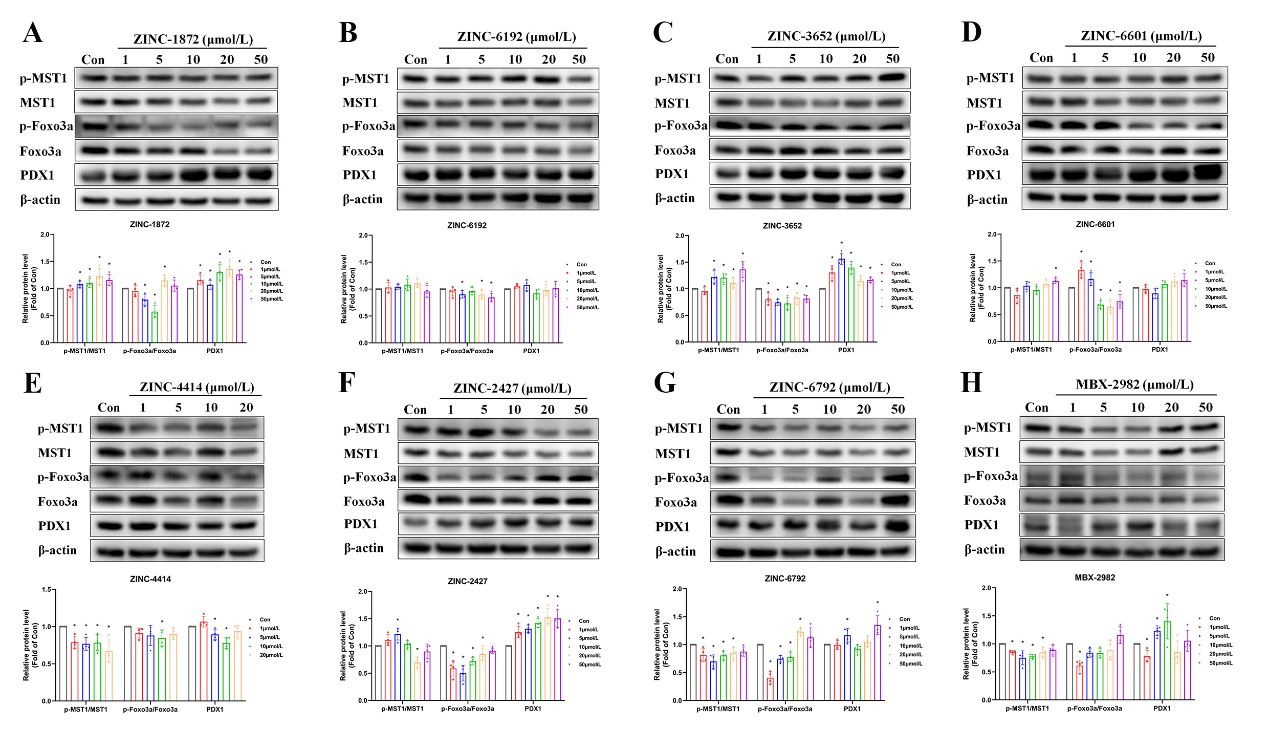


**Suppl Fig.3**


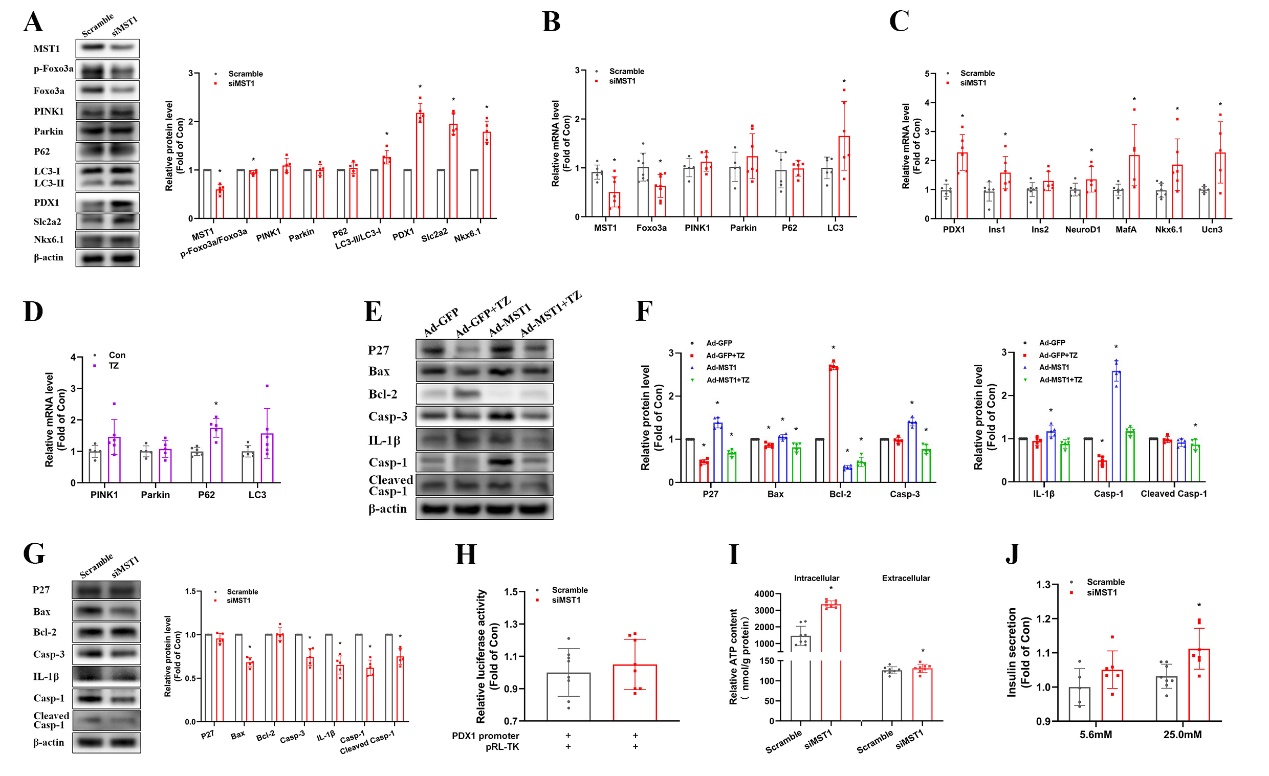


**Suppl Fig.4**


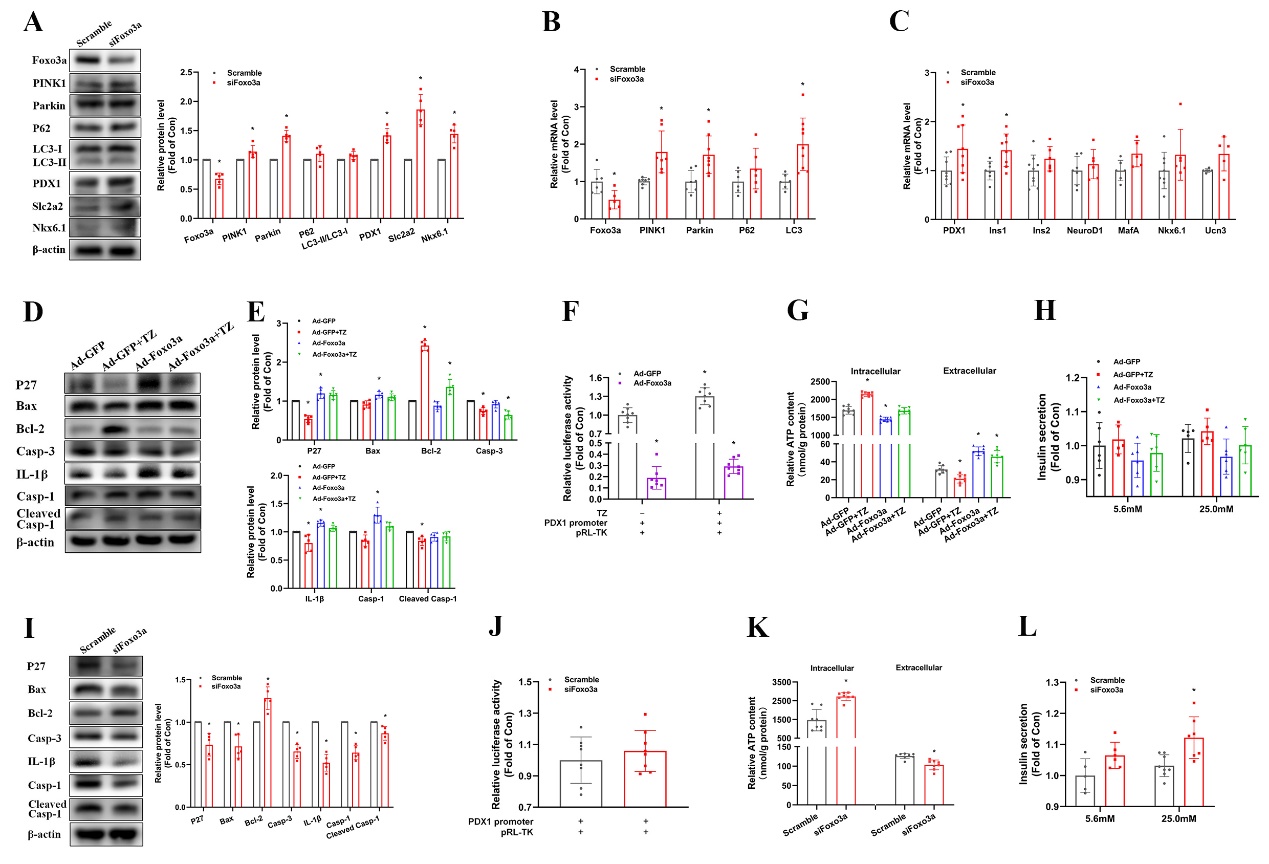


**Suppl Fig.5**


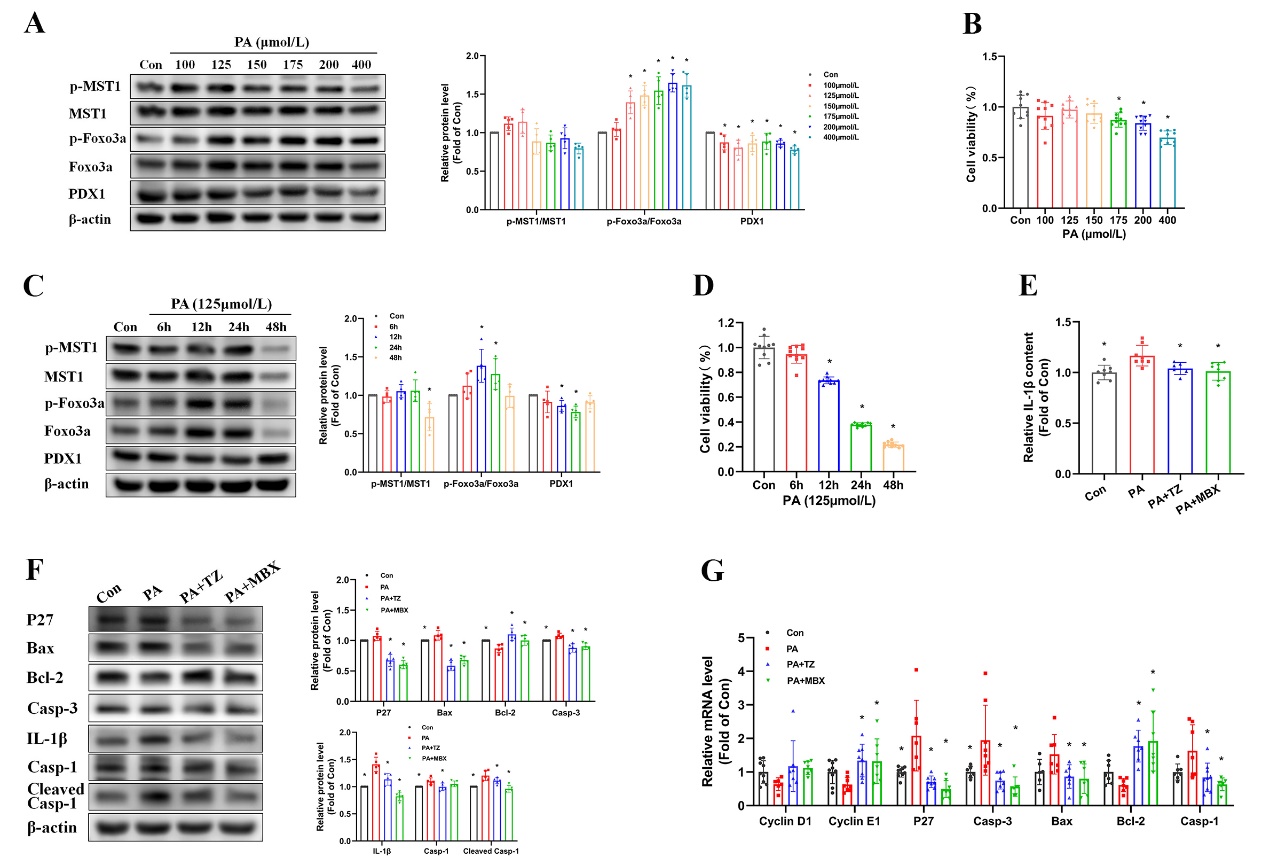


**Suppl Fig.6**


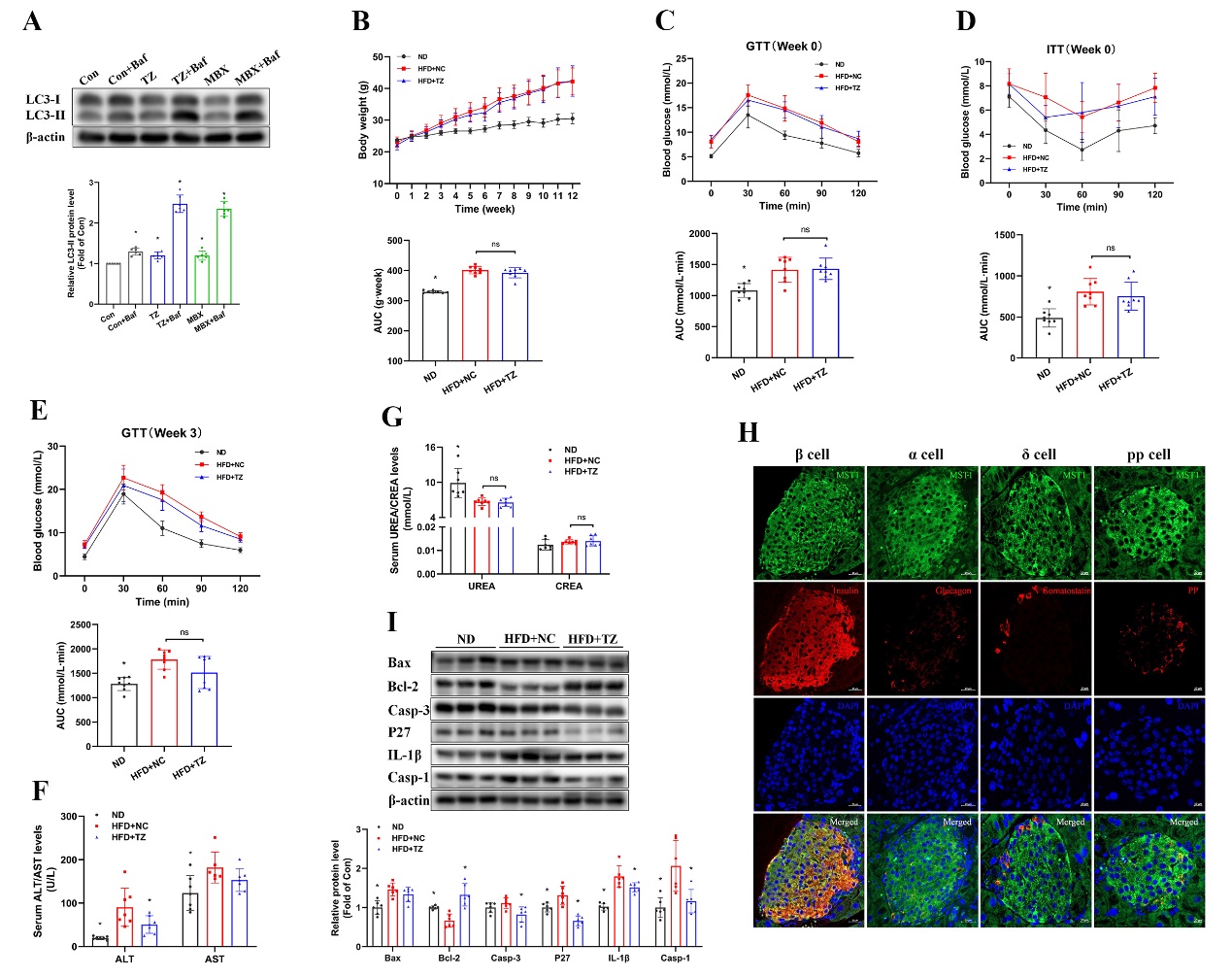


**Suppl Fig.7**


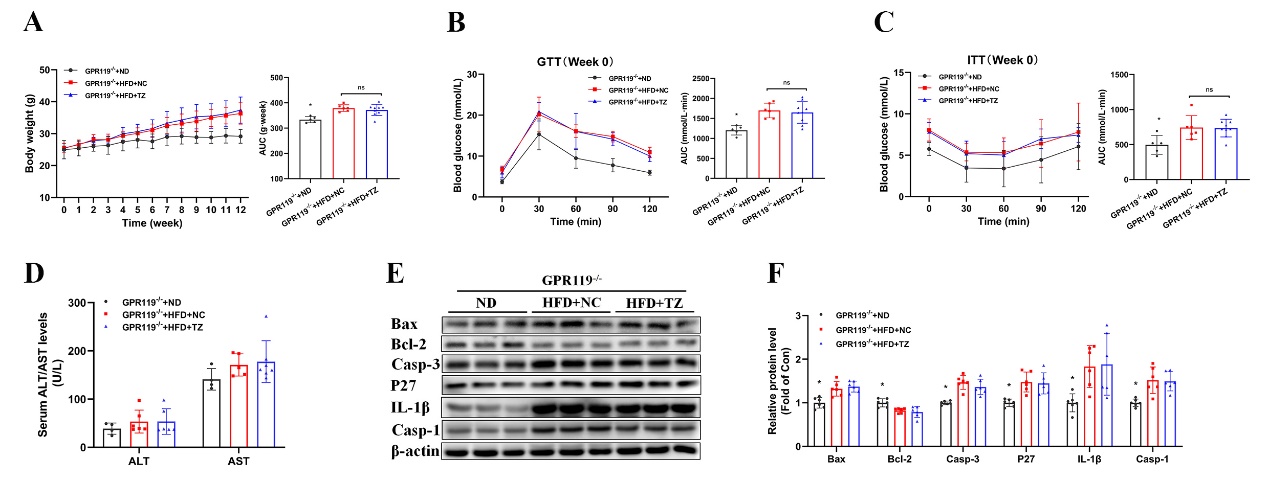


**Suppl Table 1. Primer sequences used in qRT-PCR analysis**

| Gene | Forward primer | Reverse Primer |
| --- | --- | --- |
| mouse-MST1 | GGGCTGGTTCTGTATCTGATATC | ATTTCCCGCCTTGATATCTCG |
| mouse Foxo3a | CTCACTTTGTCCCAGATCTACG | CTTCATTCTGAACGCGCATG |
| mouse PINK1 | CCTCATCTGTGCCCCTCCTG | CCAGGGACAGCCATCTGAGT |
| mouse Parkin | GGCAGTTTGTCCACGATGCT | TAATCAGGGAGTTGGGACAGCC |
| mouse P62 | CCTATACCCACATCTCCCACC | TGTCGTAATTCTTGGTCTGTAGG |
| mouse LC3 | ACAAAGAGTGGAAGATGTCCG | CCCCTTGTATCGCTCTATAATCAC |
| mouse PDX1 | CGCCACCCCAGTTTACAAGC | TGTAAGCACCTCCTGCCCA |
| mouse Ins1 | CCATCAGCAAGCAGGTCATTGT | TAGAAGAAGCCACGCTCCCC |
| mouse Ins2 | AGGACCCACAAGTGGCACAA | GGTAGGCTGGGTAGTGGTGG |
| mouse NeuroD1 | GAACCTTTTAACAACAGGAAGTGGA | CGCTCTCGCTGTATGATTTGGT |
| mouse MafA | AGGAGGAGGTCATCCGACTG | CTTCTCGCTCTCCAGAATGTG |
| mouse Nkx6.1 | GAGAGTCAGGTCAAGGTCTGGTT | CCACCGCTCGATTTGTGCTT |
| mouse Ucn3 | AGTCCACTTACAGGGAGCGAT | TGATCTGGAGGTGCGTTTGGT |
| mouse Cyclin D1 | AGTGCGTGCAGAAGGAGATT | CACAACTTCTCGGCAGTCAA |
| mouse Cyclin E1 | CCTCCAAAGTTGCACCAGTT | GGACGCACAGGTCTACAAGC |
| mouse P27 | GCGGTGCCTTTAATTGGGT | ATTCTTAATTCGGAGCTGTTTACG |
| mouse Caspase3 | CTTCATCATTCAGGCCTGCCG | GAACCACGACCCGTCCTTTG |
| mouse Bax | GATCATGAAGACAGGGGCCTTTT | CTCGATCCTGGATGAAACCCTGTA |
| mouse Bcl-2 | TGGAGAGCGTCAACAGGGAG | CACAAAGGCATCCCAGCCTC |
| mouse Caspase1 | TGCCCAGAGCACAAGACTTC | CACTCCTTGTTTCTCTCCACGG |
| mouse β actin | ACCTTCTACAATGAGCTGCG | CTGGATGGCTACGTACATGG |
